# The coupling coordination relationship and obstacle factors of public cultural services and economic development: A case study of 31 regions in China

**DOI:** 10.1371/journal.pone.0341305

**Published:** 2026-01-20

**Authors:** Bingtao Xu, Zongye Gu, Ziwen Wang, Yaqiao Chen, Yanling Wang

**Affiliations:** 1 School of Journalism and Communication, Tianjin Normal University, Tianjin, China; 2 Center for Journalism and Communication Studies, Tiangong University, Tianjin, China; National Center for Chronic and Noncommunicable Disease Control and Prevention, Chinese Center for Disease Control and Prevention, CHINA

## Abstract

The interrelationship between public cultural services (PCSs) and economic development (ED) has emerged as an important theme in academic inquiry, playing a pivotal role in advancing global sustainability. This research treats PCSs and ED as distinct systems to investigate pathways for achieving optimal coordination and balanced progress. By constructing a comprehensive evaluation framework for PCS-ED interactions, this work utilizes analytical approaches including coupled coordination measurement, kernel density analysis, spatial Markov modeling, and obstacle factor assessment to analyze their coordinated development among 31 provincial regions in China from 2014 to 2023. Our findings reveal notable geographical disparities, characterized by a pronounced positive spatial autocorrelation in coordination levels between PCS and ED. The degree of coupling coordination is higher in the eastern coastal regions, whereas that in the western and northeastern provinces exhibit lower levels of coordination. The primary impediments to balanced development are attributed to deficiencies in library exhibitions and public lectures, and constraints imposed by fiscal revenue limitations. Our findings offer valuable empirical support for optimizing the distribution of public cultural assets and fostering equitable regional economic advancement. This study echoes the global initiative of the United Nations Educational, Scientific and Cultural Organization (UNESCO) on culture for sustainable development.

## 1. Introduction

With respect to the global sustainable development agenda, the synergistic relationship between public cultural services (PCSs) and economic development (ED) has become a core issue in policy formulation and academic research. Political scientists view PCSs as important components of national governance, arguing that these services constitute the government’s fulfillment of its public service functions [1]. From a sociological perspective, PCSs are regarded as a mechanism for the redistribution of social and cultural resources aimed at promoting cultural exchange and integration among members of society [2]. Economists characterize PCSs as special types of goods with the attributes of public goods [3]. By synthesizing relevant materials, a relatively unified concept can be presented: PCSs refer to services provided by the government with the participation of social forces that have the primary objective of meeting the basic cultural needs of citizens and providing public cultural facilities, products, activities, and services to citizens [4]. PCSs cover nonprofit facilities, such as libraries, museums, cultural centers, and art galleries, to safeguard citizens’ cultural rights and improve social well-being [5]. ED involves industrial structure upgrading, increased gross domestic product (GDP) growth, and job creation [6]. According to Agenda 21 of the United Nations Statistics Division (UNSTAT), indicators for measuring ED should also consider changes in the goods trade, technology transactions, domestic consumption, industrial structure, residents’ income, and labor productivity. The interaction between PCSs and ED involves the ability to transform cultural resources into economic value, in addition to the mechanism through which economic growth feeds back into cultural development. In its “Convention on the Protection and Promotion of the Diversity of Cultural Expressions” (2005), the United Nations Educational, Scientific and Cultural Organization (UNESCO) states that PCSs are an endogenous driving force for the upgrading of the regional economic structure [7]. China’s double-cycle development strategy further positions PCSs as critical for increasing cultural consumption potential and transforming the economy [8].

The literature can be divided into two streams: theoretical research and practical research. At the theoretical level, research on the interaction between PCSs and ED originated with Western scholars’ theoretical exploration of cultural capital. Bourdieu (1986) first proposed that cultural capital influences economic output through educational inheritance and social cohesion [9]. Florida’s (2002) creative class theory reveals that there is a positive correlation between the density of cultural facilities and regional innovation capacity [10]. Alharbi (2012) offered relevant theories on the personalized provision of PCSs [11]. Wynen (2015) conducted a systematic analysis of the evolution of the PCS system and provided a theoretical foundation for its construction [12]. On the basis of Bourdieu’s theory of cultural participation, Barnes (2019) explored the impact of public participation in cocreation in PCS spaces [13]. In terms of practical research, Demetz (1970) summarized a new PCS scheme from different models applied in various countries, namely, multistakeholder cooperation, to jointly promote the supply of PCSs [14]. Alexander (2005) reported that different levels of government and state funding for PCSs have different effects on social development, industrial ecology, and other dimensions [15]. Dalle Nogare (2011) examined PCSs from the public’s perspective and argued that the frequency of public participation in cultural activities and satisfaction levels should serve as key indicators of PCS quality [16]. Greene-Moton (2020) reported that improvements in PCS standards result in high-quality EDs that benefit people, thus institutionally stimulating public enthusiasm for labor and enhancing labor productivity [17].

However, studies have focused mostly on samples of urban agglomerations in developed countries [18] and have not examined interprovincial heterogeneity in developing countries [19]. In China, PCSs are defined collectively as a government-led public service system that involves the construction of facilities and supplies activities and institutional safeguards; thus, the synergistic mechanism of PCSs with ED needs to be reexamined in the context of policy practices and spatial differences. Existing research has two shortcomings. At the theoretical level, one-way causal assumptions dominate. For example, Emilia et al. (2008) quantitatively measured the contribution of the cultural industry to GDP [20] but ignored the economic spillover effect of PCSs [1]. Polavieja (2015) constructed a two-way feedback model but did not include the spatial lag term [21], which resulted in a biased estimation. At the methodological level, the traditional coupling coordination model relies on linear weighting [22], which makes it difficult to capture the “cultural services–industrial structure–economic output” cascading effect. Although data envelopment analysis (DEA) models can be used to assess efficiency [23], closed-loop analysis and obstacle identification are not possible [24].

These deficiencies urgently need to be overcome through interdisciplinary integration and spatial heterogeneity analysis. Therefore, in this study, we take 31 provinces in China as observation units and propose the following specific research questions:

Q1: What are the evolutionary characteristics of the coupling and coordination between PCSs and ED in the spatial and temporal dimensions?
Q2: What are the barriers to the synergistic mechanisms between PCSs and ED at the interprovincial scale?

**Fig 1:**
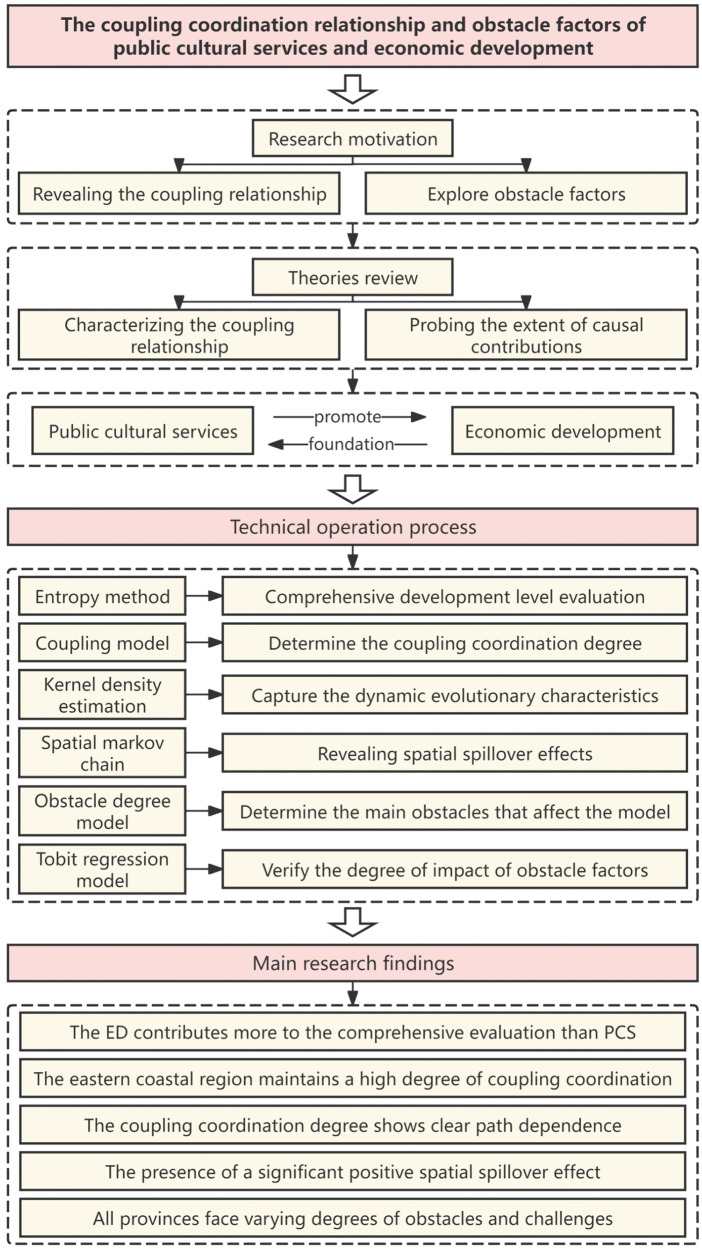
The logical framework of China’s PCS–ED integrated system.

To answer these questions, we go beyond the traditional research paradigm and construct a comprehensive analytical framework coupling the two systems. The logical framework of China’s PCS–ED integrated system is shown in Fig 1. The results provide empirical evidence for optimizing the allocation of public cultural resources and promoting balanced regional ED in echoes the global initiative of the United Nations Educational, Scientific and Cultural Organization (UNESCO) on culture for sustainable development [25].

## 2. Methodology

### 2.1 Selection of indicators

This study focuses on coupled and coordinated development between PCSs and ED. Thus, a PCS–ED indicator system was constructed to comprehensively evaluate these factors. There remains no unified indicator system, and most scholars develop their own indicator system according to their research needs.

The development of the evaluation framework adheres to key tenets including scientific rigor, inclusiveness, and practical feasibility. Through extensive literature review and analysis of PCSs definitions, we categorize the PCSs framework into three core dimensions: infrastructure for public culture, scope of public cultural resources, and public cultural programming. The subordinate indicators are formulated based on the *National Basic Public Service Standards 2023* and relevant provisions in China’s PCS legislation. Specifically, the assessment of public cultural infrastructure incorporates metrics such as amount of museum, quantity of public libraries, and availability of performance venues. The evaluation of public cultural resource scale involves measurements like library floor space per 10,000 residents and average library holdings per capita. Meanwhile, public cultural programming is gauged through indicators including exhibition frequency in public libraries, lecture series organized by libraries, and fundamental display exhibitions in museums. These indicators collectively capture operational details across museums, libraries, and art galleries, offering a holistic representation of various PCS components.

With respect to Agenda 21 published by the United Nations Statistics Division (UNSD) and Agenda 21 formulated by the Chinese government, we divide the ED system into three primary indicators: the scale of the ED, the structure of the ED, and the quality of the ED. The supplementary indicators are sourced from the China Socio-Economic Development Index Framework and the Fundamental Statistical Guidelines issued by China’s National Bureau of Statistics. Specifically, ED scale is assessed through metrics such as gross domestic product, aggregate consumer goods retail sales, and general fiscal revenue. The structural aspects of ED are evaluated by examining the tertiary industry’s share in the economy and its corresponding value-added contribution. Regarding ED quality assessment, key parameters comprise GDP per capita, urban residents’ average disposable income, and rural inhabitants’ per capita disposable income (Table 1). These indicators cover the goods trade, technology transactions, domestic consumption, and industrial structure and focus on changes in residents’ income, employment quality, and labor productivity. With this framework, we strive to comprehensively reflect the main aspects of ED.

**Table 1:** PCS–ED evaluation index system and weights.

System	Dimension	Index (unit)	Code	Weights
Public Culture Service	Facilities	Number of museums (No.)	P1	0.1061
		Number of public libraries (No.)	P2	0.0868
		Number of art performance venues (No.)	P3	0.1373
	Scale	Floor area of public libraries per 10,000 people (square meters)	P4	0.0997
		Per capita collection of public libraries (volumes)	P5	0.1351
	Activity	Number of exhibitions held by public libraries (No.)	P6	0.1641
		Number of various lectures organized by public libraries (No.)	P7	0.1478
		Number of basic display exhibitions in museums (No.)	P8	0.1231
Economic Development	Scale	Total import and export value of goods (100 million USD)	E1	0.2365
		Total retail sales of consumer goods (100 million CNY)	E2	0.0978
		Technology market transaction volume (10,000 CNY)	E3	0.2626
	Structure	Proportion of the tertiary industry (%)	E4	0.0395
		Value added of the three major industries (100 million CNY)	E5	0.0919
	Quality	Per capita gross domestic product (CNY)	E6	0.0748
		Per capita disposable personal income (CNY)	E7	0.0595
		Proportion of the urban population (%)	E8	0.0169
		Number of new urban employment jobs (10,000 people)	E9	0.0664
		Social labor productivity (10,000 CNY per person)	E10	0.0541

### 2.2 Selection of methods

In this study, we establish a PCS–ED evaluation model. We adopt six methods to support the scientific validity of the evaluation.

#### 2.2.1 Comprehensive development level evaluation model.

The two PCS and ED systems are independent and interact with each other. The comprehensive evaluation model includes 18 secondary indicators for the two systems, and objective weights need be assigned to the indicators. Research indicates that the entropy value approach serves as an effective tool for quantifying variations among different indicators. This methodology transforms both the disparities and significance levels of various metrics into numerical representations, thereby establishing a robust foundation for conducting comprehensive assessments [26].

The first step in this process is to standardize the data. As shown in Table 1, the two systems contain a total of 18 secondary indicators. To address variations in measurement units and scale magnitudes across indicators, normalization is imperative to minimize their potential influence on analytical outcomes [27]. Equation (1) is the standardization equations used


$$Y_{ij}=\frac{y_{ij}-min(y_{ij})}{max(y_{ij})-min(y_{ij})}+\text{0}.\text{0}\text{1}$$ (1)

Equation (1) is used to standardize the data through the extreme value method. In this equation, *Y*_*ij*_ represents the data of the *j*-th indicator in the *i*-th year after the standardization process, *y*_*ij*_ is the original data of the *j*-th indicator in the *i*-th year, and *max*(*y*_*ij*_) and *min*(*y*_*ij*_) identify the maximum and minimum values of the *j*-th indicator in the *i*-th year, respectively. For data with zero values, we refer to the relevant literature and uniformly add 0.01 for processing, which can prevent the assigned values from being meaningless [28].

In the second step, we calculate the information entropy of the indicator [29].


$$H_{j}=-(\frac{\text{1}}{lnn})\cdot \sum_{i=\text{1}}^{n}c_{j}\;ln\;c_{j}$$ (2)

Equation (2) is used to calculate the entropy value of a secondary indicator, where *H*_*j*_ represents the information entropy of the *j*-th indicator. *n* is the number of samples; *c*_*j*_ is the proportion of the *j*-th indicator in the *j*-th sample; and *ln* is the natural logarithm, which is used to convert probability into a measure of information quantity.

In the third step, we calculate the information utility value [30].


$$\varphi_{j}=\frac{d_{j}}{\sum_{j=\text{1}}^{m}d_{j}};d_{j}=\text{1}-H_{j}$$ (3)

Equation (3) is used to calculate the weight of a secondary indicator in this system. When the information utility value is higher, the importance of the indicator is greater in the overall evaluation. In this equation, *φ*_*j*_ represents the information utility value of the *j*-th indicator, and *m* is the total number of indicators. *d*_*j*_ represents the difference coefficient of the *j*-th indicator, and *H*_*j*_ is the information entropy of the *j*-th indicator.

In the fourth step, we calculate the indicator weights [31].


$$W_{n}=\sum_{j=\text{1}}^{m}\varphi_{ij}Y_{ij}$$ (4)

The weighting methodology employs Equation (4) to determine the relative importance of each assessment criterion, expressed as *W*_*i*_ (where *i* ranges from 1 to *k*, with *k* representing the total number of indicators). A higher weight value signifies that the corresponding indicator exerts greater influence on the ultimate evaluation outcome, thereby reflecting its heightened significance within the analytical framework.

In the fifth step, we calculate the combined evaluation level of the two systems.


$$U_{n}=\sum_{i=\text{1}}^{m}W_{ij}Y_{ij},\;\;n=\text{1},\text{2},\;\;\sum_{i=\text{1}}^{m}W_{ij}=\text{1}$$ (5)

Equation (5) provides the integrated assessment result for the evaluation criteria, derived by aggregating multiple metrics into a composite score using weighted averaging techniques. The variable *U*_*n*_ denotes the overall evaluation score for the *n*-th subject, calculated through the summation of standardized indicator values (*Y*_*ij*_) multiplied by their respective weighting coefficients (*W*_*ij*_). Specifically, *U*_*1*_ and *U*_*2*_ correspond to the synthesized evaluation outcomes for PCSs and ED systems respectively.

#### 2.2.2 Coupling coordination degree model.

The PCS and ED systems are closely linked. Therefore, the degree of coupling is utilized to explore the dynamics in terms of their interaction and interdependence. The degree of coupling reveals the degree of interaction between the two systems and is expressed as follows:


$$C=\frac{\text{2}\sqrt{U_{\text{1}}U_{\text{2}}}}{U_{\text{1}}+U_{\text{2}}}$$ (6)

Equation (6) shows the coupling degree model of the two systems [32], where C represents the coupling degree of the PCS and ED systems studied in this paper and *U*_*1*_ and *U*_*2*_ are the comprehensive evaluation indices of the PCS and ED systems, respectively.

Because the two systems differ in terms of their development, they may have a low level of development but a high degree of coupling. To prevent such a phenomenon, we introduce the coupling coordination degree model and refer to the related literature to adjust it [33].


$$D=\sqrt{C\times T},\;\;T=aU_{\text{1}}+bU_{\text{2}}$$ (7)

Equation (7) is the coupling coordination degree model, where *D* represents the coupling coordination degree of the PCS–ED system, *C* is the coupling degree of the two systems, and *T* is the comprehensive evaluation index of the two systems, which reflects the overall effectiveness or level of the combined PCS–ED system. *a* and *b* are the parameters to be estimated, and their sum is 1. Since the PCS and ED systems have the same importance [34], in the design of the pending parameter weights, we assign *a* = 0.5 and *b* = 0.5.

This study adopts the coupling coordination degree classification method proposed by Zhang et al. (2023) [35], which divides the coordination degree values into 10 levels and 4 types. This classification scheme has been widely validated in regional coupling coordination research (Table 2) [35]. Considering the uneven regional development in China, the basic coordination threshold of 0.5 is used to distinguish the coordination levels across the eastern, central, and western regions, aligning with the regional development goals outlined in the “14th Five-Year” Plan for Public Cultural Service System Construction [36].

**Table 2:** Classification of the coupling coordination degree and coupling type.

Numerical range	Coupling coordination degree	Type	Numerical range	Coupling coordination degree	Type
0.0-0.0999^*^	Extreme discordance	Lower level	0.5-0.5999	Basic coordination	High level
0.1-0.1999	Serious discordance		0.6-0.6999	Primary coordination	
0.2-0.2999	Moderate discordance		0.7-0.7999	Intermediate coordination	
0.3-0.3999	Mild discordance	Low level	0.8-0.8999	Good coordination	Higher level
0.4-0.4999	Near discordance		0.9-1.0	Excellent coordination	

* The numerical range of the extreme is 0.0–0.0999, which indicates it is infinitely close to 0.1 but does not include it.

#### 2.2.3 Kernel density estimation.

Analyzing only the spatiotemporal distribution patterns of the degree of coupling coordination does not allow for the quantitative identification of potential polarizations and disparities. Therefore, in this study, kernel density estimation (KDE) is introduced to further capture the dynamic evolutionary characteristics of the degree of coupling coordination. KDE is a nonparametric technique for estimating the probability density function of a variable and can effectively reveal the dynamic evolution of random variables [37]. It is widely applied to visualize data distributions and test hypotheses.

For a random variable *X*, the probability density function based on the kernel density estimation can be expressed as follows:


$$f_{X}(x)=\frac{\text{1}}{nh}\sum_{i=\text{1}}^{n}K\left(\frac{x-x_{i}}{h}\right)$$ (8)

In Equation (8), *n* denotes the sample size, *x*_*i*_ represents an observation of the random variable *X*, *h* refers to the bandwidth parameter, and *K*(·) denotes the kernel function. In this study, the Gaussian kernel function is employed for analysis [38].

The bandwidth *h* is a parameter in the kernel density estimation that influences the degree of smoothness of the resulting graph. A bandwidth that is too small can lead to excessive fluctuations in the graph, whereas a bandwidth that is too large may overlook important data patterns. This study follows Silverman’s rule of thumb to compute a bandwidth *h* that minimizes the asymptotic mean squared error (AMSE) using statistical measures and the sample size [39]. The equation is as follows:


$$h=\text{1}.\text{0}\text{6}\times sd\times n^{\left(-\text{1}/\text{5}\right)}$$ (9)

In Equation (9), 1.06 is the constant corresponding to the Gaussian kernel. The Gaussian kernel is typically chosen as the default because of its desirable smoothness properties. The term “*sd*” refers to a robust standard deviation designed to mitigate the influence of outliers. The factor *n*^(−1/5)^ is the sample size adjustment; as the sample size *n* increases, the bandwidth *h* decreases, and the exponent of (−1/5) represents the asymptotically optimal order derived theoretically, ensuring that the AMSE is minimized.

#### 2.2.4 Spatial Markov Chain.

A spatial Markov chain is an extension of the traditional Markov chain and is designed to overcome its limitations in geographical applications [40]. In this study, the spatial Markov chain is employed to more accurately reveal the dynamic evolutionary patterns of the degree of PCS–ED coupling coordination across 31 provinces in China, as well as its interactions with spatial factors. Unlike conventional methods, the spatial Markov chain decomposes the transition probability matrix into multiple *k* × *k* submatrices, expressed as follows:


$$M=\left(M_{ij}\right)=\left(\frac{n_{ij}}{n_{i}}\right)=(\begin{array}{ccc} n_{\text{1}\text{1}} & \cdots & n_{\text{1}j}\\ \vdots & \ddots & \vdots \\ z_{i\text{1}} & \cdots & z_{ij} \end{array})k\times k$$ (10)

In Equation (10), *M*_*ij*_ denotes the spatial transition probability. Specifically, it indicates that a spatial unit with a coupling coordination level of type *i* at time *t* transitions to type *j* at time *t* + 1. The magnitude of the spatial lag term determines the spatial lag type of the unit, with the specific expression as follows:


$$Lag=\sum_{i=\text{1}}^{n}p_{i}q_{ij}$$ (11)

In Equation (11), *Lag* denotes the spatial lag value of a region, *n* represents the number of units, *p*_*i*_ refers to the attribute value of a spatial unit, and *q*_*ij*_ indicates the spatial weight. In this study, the values of *q*_*ij*_ are derived from the geographic contiguity matrix of China’s 31 provinces: the value is set to 1 if two provinces are adjacent and 0 otherwise.

#### 2.2.5 Obstacle degree model.

To enhance the synergistic coordination of the PCS–ED integrated modeling framework, it is essential to identify the primary factors that hinder system performance. For this objective, the concept of indicator deviation is incorporated into the obstacle assessment model [41].

The first step is the calculation of the indicator deviation:


$$Z_{ij}=\text{1}-Y_{ij}$$ (12)

The second step is the calculation of the degree of the obstacle:


$$O_{i}=\frac{Z_{ij}W_{i}}{\sum_{j=\text{1}}^{n}Z_{ij}W_{i}}\times \text{1}\text{0}\text{0}\%$$ (13)

In Equations (12) and (13), *Z*_*ij*_ represents the indicator deviation and indicates the deviation value of the *i*-th indicator at the *j*-th time point or under the *j*-th condition; *Y*_*ij*_ denotes the standardized value of the data. By calculating the deviation, we can identify which indicators deviate from the expected standards or targets, thus recognizing potential anomalies. *O*_*i*_ is the obstacle degree of the indicator, which represents the obstacle degree of the *i*-th factor; *W*_*i*_ is the weight of the indicator; and *n* is the total number of samples. By calculating the obstacle degree of each factor, we can clearly identify which factors have a greater impact on the overall situation, thus providing a basis for decision making.

#### 2.2.6 Tobit regression model.

Since the degree of coupling coordination is within the range [0,1], it is a restricted dependent variable. If the ordinary least squares (OLS) estimation method was applied, the results might be biased. To more accurately quantify the strength and direction of the effects of various influencing factors on the core variable, a Tobit regression model was employed for regression analysis in the present study [42]. The model is constructed as:


$$D_{it}=\alpha_{\text{0}}+\beta_{\text{1}}{P\text{5}}_{it}+\beta_{\text{2}}{P\text{6}}_{it}+\beta_{\text{3}}{P\text{7}}_{it}+\beta_{\text{4}}{E\text{1}}_{it}+\beta_{\text{5}}{E\text{3}}_{it}+\theta_{it}$$ (14)

In Equation (14), *D*_*it*_ denotes the degree of coupling coordination. *α*_*0*_ is the constant term of the regression model. *P*5_*it*_ refers to the per capita collection of public libraries, *P*6_*it*_ indicates the number of exhibitions held by public libraries, *P*7_*it*_ represents the number of lectures organized by public libraries, *E*1_*it*_ denotes the total import and export value of goods, and *E*3_*it*_ is the transaction volume of the technology market. *θ*_*it*_ is the random disturbance term, which incorporates measurement errors and other stochastic factors. *β*_1_, *β*_2_, *β*_3_, *β*_4_ and *β*_5_ are the parameters to be estimated, and the effects of the five influencing factors on the degree of coupling coordination are measured. *i* represents city *i*, and *t* denotes year *t*.

### 2.3 Data sources

The research encompasses 31 provincial-level administrative divisions in China, excluding Hong Kong SAR, Macao SAR, and Taiwan due to incomplete datasets for certain variables. In compliance with scientific data collection standards, the PCS-ED metrics were obtained from authoritative sources including the *China Statistical Yearbook*, *China Cultural and Related Industries Statistical Yearbook*, *China Economic Census Yearbook*, and the EPS database. We ensure that any third-party data licensing terms permit redistribution. Considering data accessibility, this investigation utilized survey information spanning from 2014 to 2023. Due to historical and geographical issues, data from Tibet Autonomous Region after 2020 is missing. We use interpolation to fill in the missing observations [43]. For original data, please refer to *https://doi.org/10.3886/E239182V2*.

## 3. Results analysis

### 3.1 Analysis of the comprehensive development level

**Fig 2:**
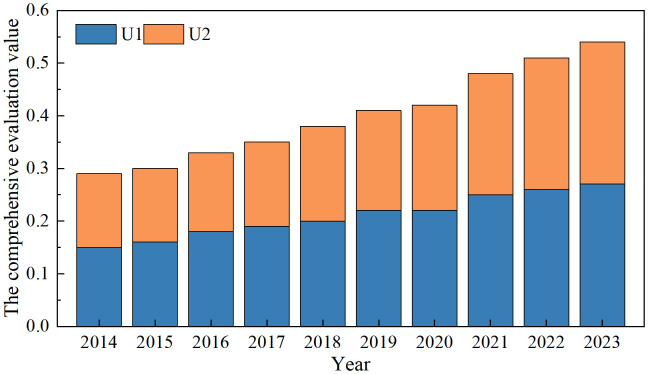
The comprehensive evaluation value of PCSs and ED.

We use SPSS 27.0 to calculate the comprehensive evaluation values and coupling coordination degree of the PCS and ED systems in 31 provinces across China. To clearly evaluate the relationship between PCSs and ED in China from 2014 to 2023, we use Origin 2024 to visualize the comprehensive evaluation values of the two systems, and the results are presented in Fig 2. As shown in Fig 2, the overall comprehensive evaluation value of the PCSs tends to increase from 0.1460 in 2014 to 0.2724 in 2023. The comprehensive evaluation value of ED also shows a clear increasing trend, increasing from 0.1380 in 2014 to 0.2673 in 2023. The growth rate of the ED value is slightly greater than that of the PCS value. These findings indicate that compared with PCSs, ED contributes more to the comprehensive evaluation, but the importance and scale of PCSs are also increasing. This study takes P1 and E1 as examples and calculates the comprehensive evaluation value of these two indicators step by step, based on the aforementioned equations. The detailed calculation process is provided in S1 File.

**Fig 3:**
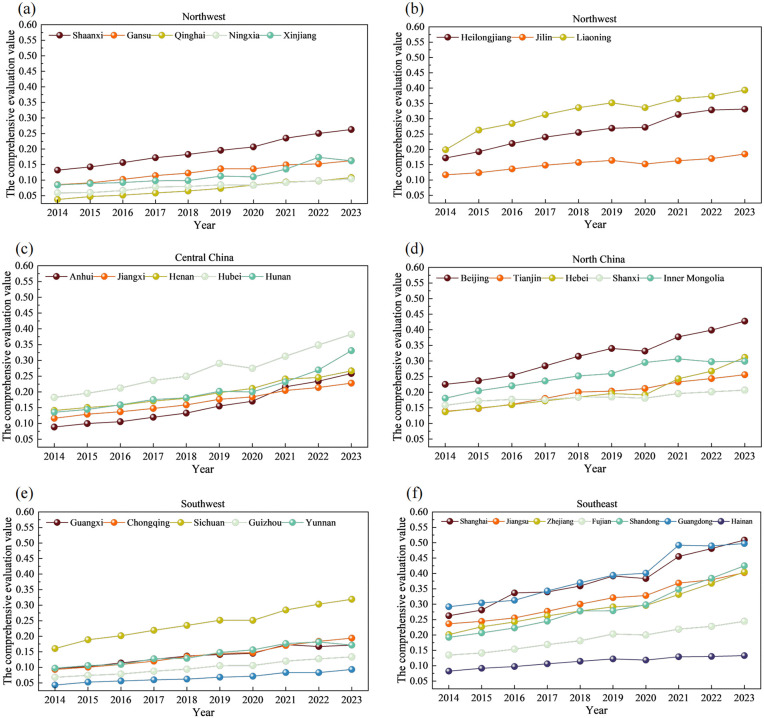
Comprehensive development level of each region.

We divide China’s 31 provinces into six regions according to their geographical orientation [44] to obtain a more intuitive understanding of the regional differences in the integrated development of the two systems (Table 3). The changes in the level of comprehensive development of each region are shown in Fig 3.

**Table 3:** Regional division of 31 provinces in China.

Regions	Provinces	Regions	Provinces
Northwest	Shaanxi	Northeast	
	Gansu		Heilongjiang
	Qinghai		Jilin
	Ningxia		Liaoning
	Xinjiang		
Central China	Anhui	North China	Beijing
	Jiangxi		Tianjin
	Henan		Hebei
	Hubei		Shanxi
	Hunan		Inner Mongolia
Southwest	Guangxi	Southeast	Shanghai
	Chongqing		Jiangsu
	Sichuan		Zhejiang
	Guizhou		Fujian
	Yunnan		Shandong
	Tibet		Guangdong
			Hainan

Notes: Because of missing data, Hong Kong, Macau, and Taiwan were not included in the present study.

Across all the regions, the comprehensive evaluation values show an overall increasing trend, although provincial differences remain evident. In the northwest (Fig 3a), Shaanxi ranks highest because of its strong public cultural facilities and activities (P1-3, P6-8) and ED scale and quality (E1-3, E5, E10), whereas Qinghai scores lowest. Post-2020 fluctuations were driven by increasing library activities (P2, P6, P7) and ED quality (E6, E7, E9) in Xinjiang. In the northeast (Fig 3b), Liaoning performs well in terms of the PCS scale and activities and most ED indicators; Jilin lags in nearly all indicators, and Heilongjiang shows moderate growth across most indicators. In Central China (Fig 3c), Hubei Province scores highly across various indicators, including public cultural facilities and activities (P1-3, P6-8) and ED quality (E6, E8, E10). Notably, from 2020 to 2023, the growth rates of the comprehensive development levels of Hubei, Hunan, and Anhui accelerated, with the slopes of their growth curves increasing. In North China (Fig 3d), the ED indicators of Beijing performed well, with all the indicators scoring high. Inner Mongolia scored high on the PCS indicators. After 2020, Hebei’s comprehensive development level increased significantly faster. The data indicate that these results are closely related to improvements in the scores for indicators such as PCS activities (P6, P7) and the number of new urban jobs (E9). In the southwest (Fig 3e), Sichuan displays the most prominent growth across all the indicators, with relatively high starting values and rapid growth. Tibet’s scores for all the indicators have remained consistently low over the years, which is the key factor contributing to its relatively low overall development level. In the southeast (Fig 3f), Shanghai and Guangdong dominate, with the former excelling in PCSs and the latter in ED. Fujian and Hainan grew at slower rates, with Hainan constrained by persistently low ED performance.

### 3.2 Analysis of the degree of coupling coordination

#### 3.2.1 Temporal evolution analysis.

**Fig 4:**
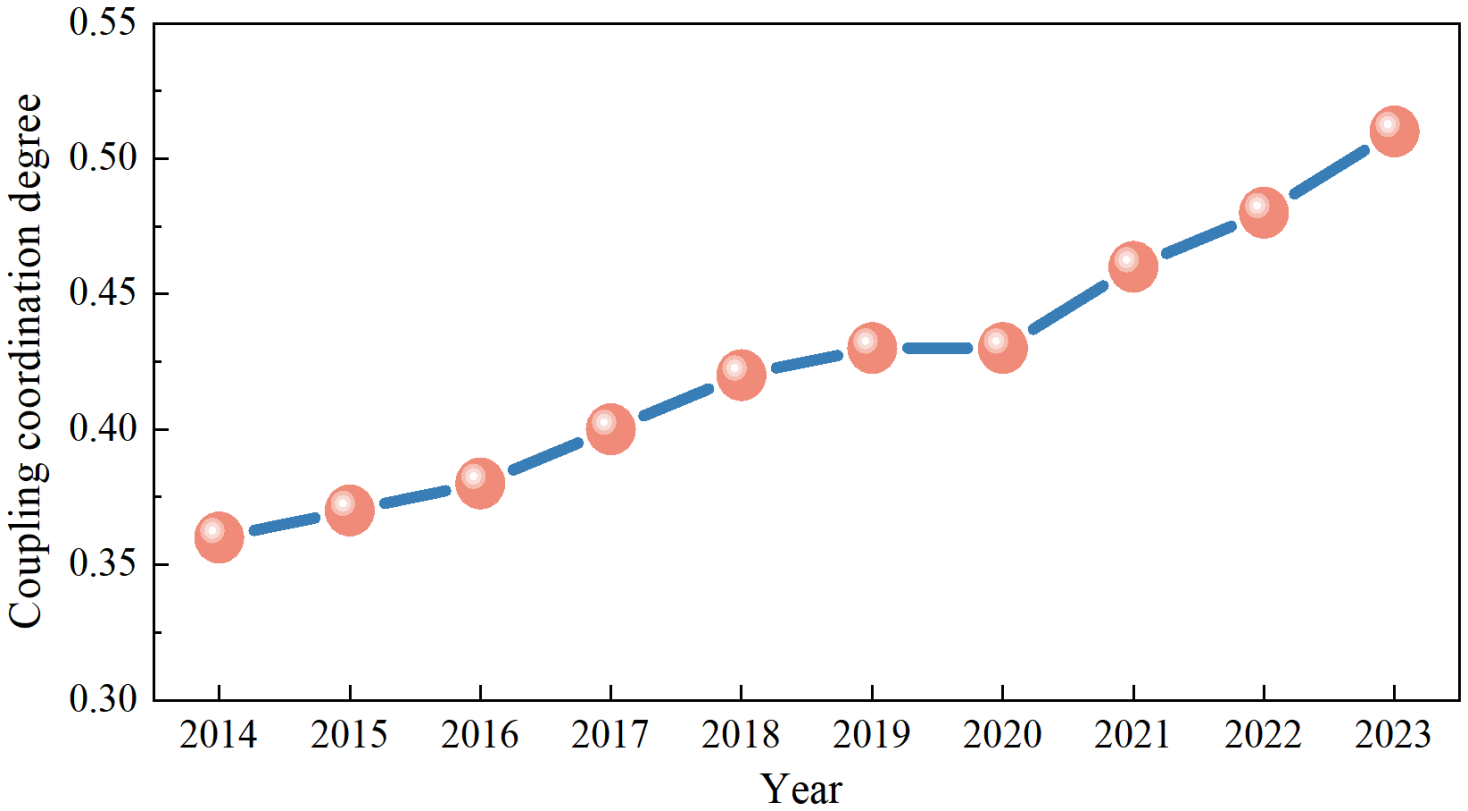
Degree of coupling coordination of PCSs and ED.

According to the data on the degree of coupling coordination between PCSs and ED in China’s 31 provinces from 2014 to 2023, the degree of coupling coordination steadily increased (Fig 4). The degree of coupling coordination was 0.3575 in 2014 and increased to 0.5112 in 2023. These findings show that the synergistic relationship between China’s PCSs and ED has been optimized over the past ten years, and the relationship has gradually developed from a low to a high level of coordination.

The data in Fig 4 indicate that from 2014 to 2023, the degree of coupling coordination between China’s PCSs and ED presented a three-phase characteristic. From 2014 to 2017, the degree of coupling coordination increased from 0.3575 to 0.4017, with a relatively rapid growth rate. From 2017 to 2020, the degree of coupling coordination increased from 0.4017 to 0.4332, and the growth rate began to slow. From 2020 to 2023, the degree of coupling coordination increased from 0.4332 to 0.5112, with a rapid growth rate and a steep slope. With the state’s increasing emphasis on the construction of PCS systems, the connection between PCSs and economic growth has increased, and the effects of resource sharing and complementary advantages have begun to emerge.

As shown in Fig 4 and Table 2, China’s PCS–ED coupling coordination transitioned from a low to a high level from 2014 to 2023. From 2014 to 2016, it was in the mild coordination stage; from 2017 to 2022, it was in the near-basic coordination stage; and in 2023, it entered the basic coordination stage. This indicates that although China’s PCSs and ED have made great progress in terms of coordinated development, there is still much room for improvement, and the future goal is to move toward a high degree of coordination.

#### 3.2.2 Spatial evolution analysis.

**Fig 5:**
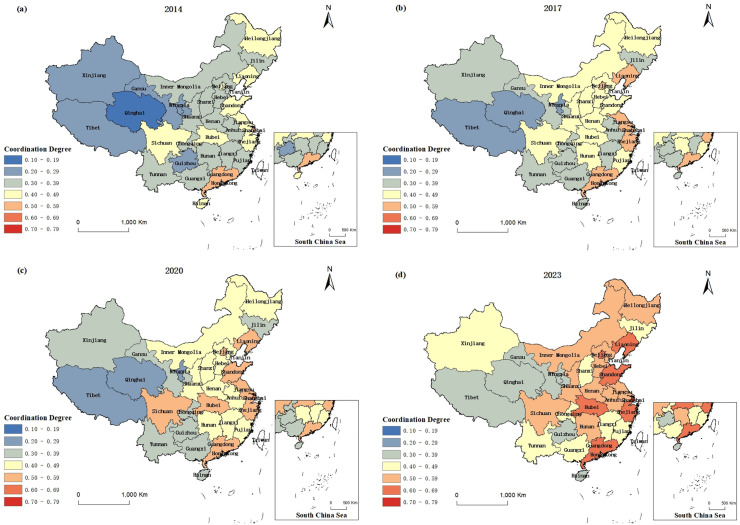
Spatial evolution of the coupling coordination of PCSs and ED. Notes: The authors declare that no copyrighted figures have been used in this manuscript. Fig 5 is based on the standard map with review number GS (2020)4619 on the standard map service website of the Ministry of the Chinese Natural Resources, with no modifications to the base map. URL: http://bzdt.ch.mnr.gov.cn/download.html?searchText=GS%2520(2020)4619.

To intuitively explore the spatial evolutionary characteristics of the coupled and coordinated relationships between PCSs and ED in China, we select 2014, 2017, 2020, and 2023 as representative years. We use ArcGIS 10.8 to spatially visualize the degree of coupling and coordination of the PCS–ED system in the 31 provinces in China (Fig 5).

As shown in Fig 5, from 2014 to 2023, the spatial distribution of the degree of coupling coordination between PCSs and ED in China’s 31 provinces consistently exhibited an east–west gradient. In 2014 (Fig 5a), eastern coastal provinces such as Guangdong, Shanghai, and Shandong had relatively high levels of coordination, resulting in the formation of high-value clusters. Central provinces, including Henan, Hunan, and Jiangxi, were at medium to low levels of coordination and served as a transition zone. The western regions, such as Qinghai, Guizhou, and Tibet, exhibited low coordination. By 2017 (Fig 5b), the eastern provinces maintained their advantage, with Shanghai, Zhejiang, and Jiangsu forming high-value clusters. Sichuan, Shaanxi, and Hubei also had relatively high levels of coordination and were connected with developed eastern areas. The western provinces continued to show low levels of coordination. In 2020 (Fig 5c), the eastern provinces continued to show the highest levels of coordination, although Fujian and Hainan lagged. Strong differentiation was observed in North China, with Beijing serving as a high-value center. Trends in the central and western regions also varied. Sichuan reached basic coordination levels, whereas Qinghai, Tibet, and Ningxia continued to show low levels of coordination. By 2023 (Fig 5d), coordination improved in all regions. Eastern provinces represented core high-value areas, with the value of Shanghai exceeding 0.7 and those of Guangdong, Zhejiang, and Shandong being above 0.6. The coordination in the central region was generally moderate and led by Hubei. The coordination in the northwestern and southwestern regions was mostly low, although Shaanxi and Sichuan performed relatively well in terms of coordination.

The coupling coordination degree classification standards referenced in this study are based on Table 2, building upon previous research. To verify the sensitivity of the classification thresholds and the robustness of the conclusions, this study sets two alternative threshold groups, based on the original thresholds. The first alternative threshold (a) increases the original threshold by 0.02, while the second alternative threshold (b) decreases it by 0.02. The coupling coordination degree levels for 31 provinces in China in 2019 are recalculated using these alternative thresholds, with the results shown in Table 4.

**Table 4:** Sensitivity test results of coupling coordination threshold.

Provinces	Original threshold	Alternative threshold (a)	Alternative threshold (b)
Beijing	Basic coordination	Basic coordination	Basic coordination
Tianjin	Near discordance	Near discordance	Near discordance
Hebei	Near discordance	Near discordance	Near discordance
Shanxi	Near discordance	Mild discordance	Near discordance
Inner Mongolia	Near discordance	Near discordance	Near discordance
Liaoning	Basic coordination	Basic coordination	Basic coordination
Jilin	Mild discordance	Mild discordance	Near discordance
Heilongjiang	Near discordance	Near discordance	Near discordance
Shanghai	Primary coordination	Primary coordination	Primary coordination
Jiangsu	Basic coordination	Basic coordination	Basic coordination
Zhejiang	Basic coordination	Basic coordination	Basic coordination
Fujian	Near discordance	Near discordance	Near discordance
Shandong	Basic coordination	Basic coordination	Basic coordination
Guangdong	Basic coordination	Basic coordination	Primary coordination
Hainan	Mild discordance	Mild discordance	Mild discordance
Anhui	Mild discordance	Mild discordance	Near discordance
Jiangxi	Near discordance	Mild discordance	Near discordance
Henan	Near discordance	Near discordance	Near discordance
Hubei	Basic coordination	Basic coordination	Basic coordination
Hunan	Near discordance	Near discordance	Near discordance
Guangxi	Mild discordance	Mild discordance	Mild discordance
Chongqing	Mild discordance	Mild discordance	Mild discordance
Sichuan	Basic coordination	Near discordance	Basic coordination
Guizhou	Mild discordance	Mild discordance	Mild discordance
Yunnan	Mild discordance	Mild discordance	Mild discordance
Tibet	Moderate discordance	Moderate discordance	Moderate discordance
Shaanxi	Near discordance	Near discordance	Near discordance
Gansu	Mild discordance	Mild discordance	Mild discordance
Qinghai	Moderate discordance	Moderate discordance	Moderate discordance
Ningxia	Moderate discordance	Moderate discordance	Mild discordance
Xinjiang	Mild discordance	Mild discordance	Mild discordance

As shown in Table 4, under the alternative threshold (a), the coupling coordination levels of Shanxi and Sichuan decrease among the 31 provinces. Under the alternative threshold (b), the coupling coordination levels of Jilin and Guangdong increase. While the coupling coordination degree classification thresholds in this study result in minor adjustments in the levels of individual provinces, the overall distribution pattern of regional coupling coordination levels and the core differential characteristics remain substantively unchanged. This suggests that the conclusions regarding the coupling coordination degree classification in this study are not dependent on specific threshold settings, demonstrating the robustness and reliability of the results.

#### 3.2.3 Spatiotemporal dynamics analysis.

**Fig 6:**
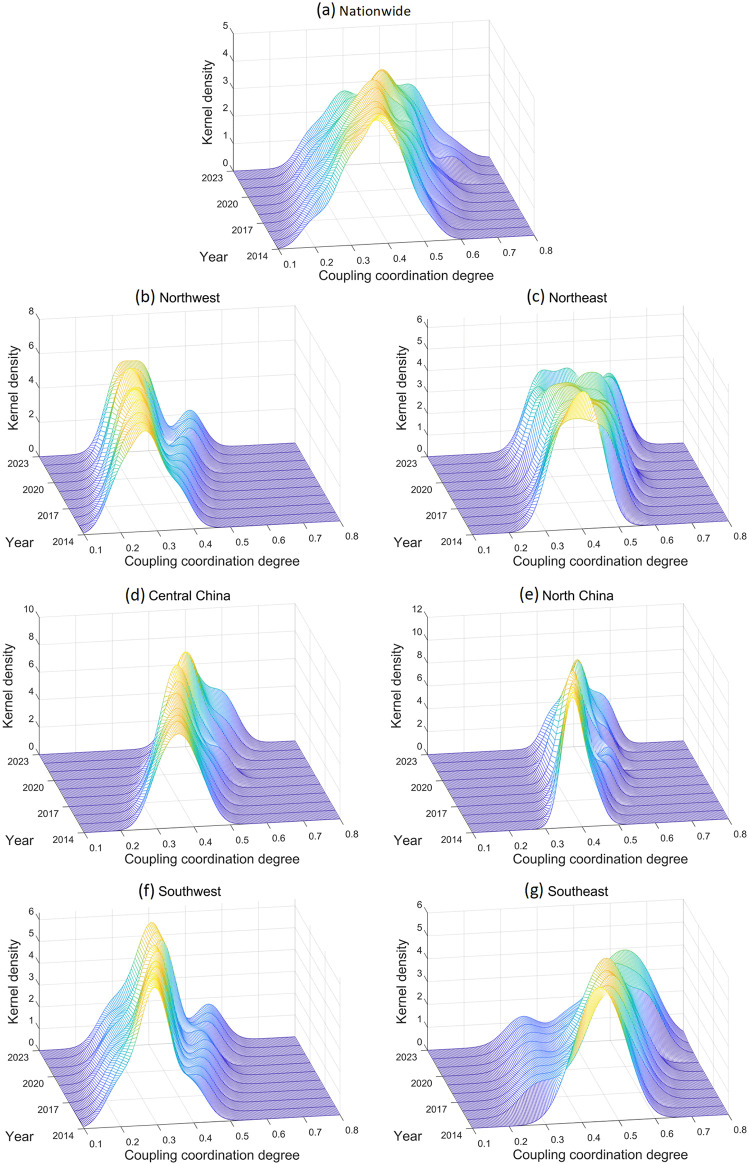
The spatiotemporal dynamics of the coupling coordination of PCSs and ED.

To more accurately characterize the dynamic evolution of coupling coordination development, this study employs MATLAB 2023 for KDE. The spatiotemporal evolution of coupling coordination across regions is analyzed in terms of location, morphology, extensibility, and polarization characteristics (Fig 6). This study conducts KDE of the coupling coordination degree for the entire country and six regional divisions. The bandwidth for each region is calculated individually using Silverman’s Rule of Thumb, based on the respective regional data, as shown in Equation (9).

As can be inferred from Fig 6a, from the perspective of the nation, the distribution of kernel density exhibits several local peaks. Fig 6a suggests that the coupled coordination degree does not concentrate on a single range, but is widely distributed among various typical ranges. Since 2014, the position and overall hight of each peak exhibit regular time patterns. The positions of the density peaks shifted to the right over time, indicating that the overall coordination between PCSs and ED across the country was gradually improving. In the early period, the kernel density curve was flatter, indicating there was large difference among regions. While the kernel density curve in the latter period was taller, which means there was little difference in different places.

In northwestern (Fig 6b) and southwestern (Fig 6f) part, kernel density curve indicates a markedly divergent interior. Multiple peaks can be found each year, indicating the uneven coupling coordination these places. In the period of 2014−2023, Fig 6b and 6f was heterogenous, and exhibits slow rate of coordination improvements. Northeast part (Fig 6c) exhibits multipeaks in density distribution, where the peaks shift to the right increase in height as time passes. Fig 6c demonstrates there is a highly dynamic and nonstationary interaction between PCSs and ED, indicating the development model of the region is changing. In Central China (Fig 6d) the degree of coordination coupling was around 0.35 for a long time. The degree of coordination coupling gradually increased to over 0.45 since 2018. The occurrence of the bimodal trend shows that there is greater differentiation between provinces in the region when it comes to coordination levels. North China (Fig 6e) remains degree of coordination coupling at an intermediate level (0.3–0.4) in general, showing a relatively stable long-term state. However, the height of kernel density primary peak declined gradually and became multifarious patterns, which indicates that regional development is not concentrated at a certain level anymore, and the provincial disparity is increasing. The southeastern part (Fig 6g) exhibits core intervals (0.4–0.5) are higher than that in North and Center China, which indicates an excellent overall coordination. Especially after 2020, kernel density distribution exhibit a more obvious multiple peak features with internal disparity and different direction of development growth. Unlike North and Central China which show only single- or double-peaks distribution, Southeast China shows a more complicated multi-peak pattern that reflects the complexity of coordinated development in different regions of Southeast China.

To verify the robustness of the bandwidth, a sensitivity plot for the neighboring bandwidth at the national level for 2019 is presented in S1 Fig. The kernel density estimate for the national level in 2019 uses a bandwidth of 0.0345, with neighboring bandwidths set at 0.0245 and 0.0445. The results show that the distribution characteristics of the coupling coordination degree remain stable across the neighboring bandwidths. This suggests that the conclusions drawn from the kernel density analysis in this study are not sensitive to the subjective selection of the bandwidth.

In this study, the spatial Markov chain is employed to more accurately reveal the dynamic evolutionary patterns of the degree of PCS–ED coupling coordination across 31 provinces in China, as well as its interactions with spatial factors. In this study, a spatial adjacency matrix for China’s 31 provinces is constructed, and a spatial Markov chain model is employed. The degree of coupling coordination between PCSs and ED in these provinces is classified into four categories—low (I), medium (II), high (III), and very high (IV)—via the quantile method. By setting a one-year lag as the condition, the transition probability matrix of the degree of coupling coordination was calculated with MATLAB 2023. The results are presented in Table 5.

**Table 5:** Transition probability matrix of the coupling coordination of PCSs and ED.

Spatial lag type	t/(t + 1)	I	II	III	IV	Observed values
I	I	0.9259	0.0741	0.0000	0.0000	27
	II	0.0000	0.8333	0.1667	0.0000	6
	III	0.0000	0.0000	0.8000	0.2000	5
	IV	0.0000	0.0000	0.0000	1.0000	3
II	I	0.8788	0.1212	0.0000	0.0000	33
	II	0.0000	0.7917	0.2083	0.0000	24
	III	0.0000	0.0417	0.7917	0.1667	24
	IV	0.0000	0.0000	0.0000	1.0000	11
III	I	0.6667	0.3333	0.0000	0.0000	9
	II	0.0000	0.7879	0.2121	0.0000	33
	III	0.0000	0.0000	0.7931	0.2069	29
	IV	0.0000	0.0000	0.0345	0.9655	29
IV	I	0.8333	0.1667	0.0000	0.0000	6
	II	0.1429	0.7143	0.1429	0.0000	7
	III	0.0000	0.0000	0.7333	0.2667	15
	IV	0.0000	0.0000	0.0000	1.0000	18

Even considering the influence of neighbors at different levels, the probabilities of the main diagonal elements in the PCS–ED coupling coordination transition matrix for China’s 31 provinces are consistently higher than those of the off-diagonal elements (Table 5). These findings indicate a pronounced path dependence across regions: under varying neighbor conditions, provinces are highly likely to remain within their original coordination level during the next period.

Further examination of transition probabilities under different neighbor-level influences reveals several regular patterns. First, for low-level (I) provinces, the probability of upgrading to the medium level (II) increases to 33.33% as neighbor levels increase. When neighbors are at the very high level (IV), this probability reaches 16.67%, which is significantly higher than the 7.41% observed when neighbors are at the low level (I). This suggests that the upgrading of low-level provinces has a stronger positive spatial association with higher-level neighbors. Second, for medium-level (II) provinces, the probability of advancing to a high level (III) reaches 21.21% when neighbors are at a high level (III), which is the highest among all the scenarios. These findings indicate that the upgrading of medium-level provinces has the most notable spatial correlation with that of high-level neighbors. Third, for high-level (III) provinces, as neighbor levels increase from medium (II) to very high (IV), the probability of advancing to a very high level (IV) increases from 16.67% to 26.67%. These findings indicate that the further upward transition of high-level provinces is associated with a notable positive spatial pattern relative to very high-level neighbors. Fourth, for very high-level (IV) provinces, when neighbors are also at a very high level (IV), the probability of remaining at the original level reaches 100%. These findings indicate that clusters of very high-level neighbors are linked to the consolidation of a province’s leading position, confirming hints of positive spatial correlation: High-quality neighbors are associated with the upgrading of lower-level provinces and are linked to the stability and advantage of higher-level provinces.

### 3.3 Analysis of the obstacle degree

#### 3.3.1 Main obstacle factors.

To further explore which factors hinder the level of coupled and coordinated PCSs and ED, we calculate the indicator deviations and obstacles using Equations (12) and (13). We use the obstacle degree model to explore the obstacles affecting the coupled and coordinated development of PCSs and ED across 31 provinces in China from 2014 to 2023. By calculating the average value of the obstacle degree of each indicator, the final obstacle degree of each province is calculated, and the top three obstacles are defined as the main obstacles (Table 6).

**Table 6:** Ranking the main obstacles of the selected provinces.

Regions	Provinces	Item	PCS system			ED system		
			Ranking of main obstacle indices (%)					
			1st	2nd	3rd	1st	2nd	3rd
North China	Beijing	Index	P6	P7	P3	E1	E3	E2
		Degree	18.47	15.34	13.75	32.86	16.38	14.82
	Tianjin	Index	P6	P7	P8	E3	E1	E2
		Degree	19.31	17.05	13.99	29.47	27.21	11.26
	Hebei	Index	P6	P5	P7	E3	E1	E2
		Degree	17.61	16.67	14.62	29.74	27.11	8.38
	Shanxi	Index	P6	P7	P5	E3	E1	E2
		Degree	19.13	16.2	15.34	28.96	25.98	9.39
	Inner Mongolia	Index	P6	P7	P3	E3	E1	E2
		Degree	18.8	16.79	15.22	29.12	25.97	9.62
Northeast China	Liaoning	Index	P6	P7	P8	E3	E1	E2
		Degree	18.32	15.26	13.86	29.15	25.89	8.93
	Jilin	Index	P6	P7	P3	E3	E1	E2
		Degree	18.22	16.31	12.81	28.35	25.69	9.61
	Heilongjiang	Index	P6	P7	P3	E3	E1	E2
		Degree	18.19	16.59	14.18	28.58	25.82	9.41
Southeast China	Shanghai	Index	P6	P7	P3	E3	E1	E2
		Degree	21.38	17.41	16.15	33.38	23.75	11.44
	Jiangsu	Index	P6	P7	P5	E3	E1	E7
		Degree	20.52	17.33	16.31	25.95	25.02	8.07
	Zhejiang	Index	P5	P6	P1	E3	E1	E5
		Degree	16.73	15.92	13.33	35.75	23.44	8.21
	Fujian	Index	P6	P7	P3	E3	E1	E5
		Degree	18.39	15.95	14.37	32.89	25.07	8.34
	Shandong	Index	P5	P6	P3	E3	E1	E6
		Degree	20.28	20.05	13.63	32.53	26.94	8.35
	Guangdong	Index	P6	P5	P3	E3	E6	E10
		Degree	17.69	16.84	16.04	35.55	11.71	9.03
	Hainan	Index	P6	P7	P3	E3	E1	E2
		Degree	17.14	15.26	13.81	26.31	25.78	10.46
Central China	Anhui	Index	P6	P5	P7	E3	E1	E5
		Degree	17.36	16.11	15.01	27.71	27.19	8.44
	Jiangxi	Index	P6	P7	P5	E3	E1	E2
		Degree	17.94	15.78	14.56	28.76	25.84	9.06
	Henan	Index	P6	P5	P7	E3	E1	E6
		Degree	19.69	18.66	14.8	31.37	27.56	8.04
	Hubei	Index	P6	P7	P5	E1	E3	E5
		Degree	17.86	16.08	14.93	29.57	26.97	8.38
	Hunan	Index	P6	P5	P7	E3	E1	E5
		Degree	18.71	15.24	14.12	28.29	27.83	8.14
Northwest China	Shaanxi	Index	P6	P7	P5	E1	E3	E2
		Degree	18.15	16.69	15.77	27.39	25.07	9.63
	Gansu	Index	P6	P7	P3	E3	E1	E2
		Degree	18.71	16.44	15.04	27.52	25.33	9.81
	Qinghai	Index	P6	P7	P3	E3	E1	E2
		Degree	17.99	16.2	14.02	27.43	24.79	10.15
	Ningxia	Index	P6	P7	P3	E3	E1	E2
		Degree	17.96	16.31	15.33	27.72	24.95	10.17
	Xinjiang	Index	P6	P7	P3	E3	E1	E2
		Degree	17.02	16.15	15.24	28.51	25.21	10.01
Southwest China	Guangxi	Index	P6	P7	P3	E3	E1	E2
		Degree	17.41	15.51	14.81	28.62	24.87	9.16
	Chongqing	Index	P6	P7	P3	E3	E1	E5
		Degree	16.89	15.22	14.03	30.27	26.03	9.14
	Sichuan	Index	P6	P5	P7	E3	E1	E6
		Degree	19.6	16.99	16.12	29.43	27.71	8.03
	Guizhou	Index	P6	P7	P3	E3	E1	E2
		Degree	17.15	15.09	14.69	28.35	25.96	9.51
	Yunnan	Index	P6	P3	P7	E3	E1	E2
		Degree	17.11	15.39	15.28	28.81	25.59	9.04
	Tibet	Index	P6	P7	P3	E3	E1	E2
		Degree	17.98	16.28	14.38	27.33	24.61	10.11

Notes: P1 represents the number of museums, P3 represents the number of art performance venues, P5 represents the per capita collection of public libraries, P6 represents the number of exhibitions held by public libraries, P7 represents the number of various lectures organized by public libraries, and P8 represents the number of basic display exhibitions in museums. E1 denotes the total import and export value of goods, E2 denotes the total retail sales of consumer goods, E3 denotes the technology market transaction volume, E5 denotes the value added of the three major industries, E6 denotes per capita gross domestic product, E7 denotes per capita disposable personal income, and E10 denotes social labor productivity. Please refer to Table 1 for details.

Table 6 presents the top three barriers and their respective proportions for the PCS–ED system across 31 provinces in China from 2014 to 2023. In terms of the obstacles in the PCS system, P6 and P7 are the primary obstacles in most provinces, indicating that libraries generally lack capabilities in the planning, organization, and promotion of exhibitions and lectures. P5 has become an obstacle in multiple provinces, which indicates that the book collections of some libraries cannot meet the reading and learning needs of the public. P3, P8, and P1 are obstacles in some provinces and identify shortcomings in the number of art performance venues and museum exhibition resources, hindering the diversified development of PCSs. From the perspective of the obstacles in the ED system, E1, E2, and E3 are identified as obstacles in more than half of the provinces, suggesting that factors such as the goods trade, consumer markets, and technology markets affect ED. Additionally, E5, E6, and E10 are identified as obstacles in some provinces, which reveals that the quality of ED in these provinces needs to be improved.

**Fig 7:**
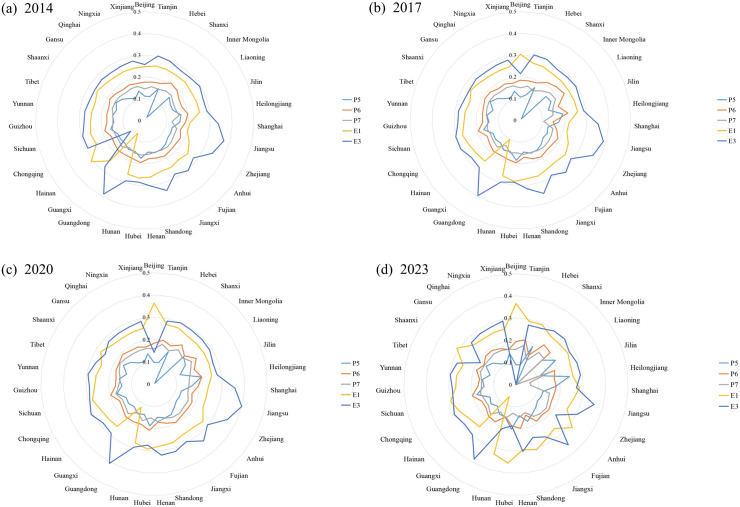
Obstacle analysis of China’s PCS–ED system.

To more clearly show the changes in the level of obstacles in each province from 2014 to 2023 and determine whether the obstacle levels in each province change over time, we calculate the obstacle levels in the 31 provinces over the same period for 2014, 2017, 2020, and 2023 and retain the top five obstacles as the main obstacles (Fig 7).

As shown in Fig 7, most provinces’ obstacle index values for the ED system fall within the outer ring of the radar chart. Both E1 and E3 present relatively high levels of obstacles, indicating that most provinces face challenges in terms of their ED scale. From 2014 to 2023, the obstacle index value for the total import and export value of goods (E1) slightly increased. Beijing and Hubei ranked among the top three provinces in multiple years and maintained relatively high obstacle index values. The obstacle degree of the technology market transaction volume (E3) fluctuated slightly but remained at a high level throughout the period. Guangdong, Jiangsu, Zhejiang, and Fujian Provinces consistently ranked among the top three in all years, indicating that these regions faced significant obstacles in technology market transactions. Most provinces have obstacle scores for the public cultural system within the inner ring of the radar chart. The obstacle score for P6 is higher than those for P5 and P7, which implies that most provinces face certain obstacles in terms of the public library scale and activities. From 2014 to 2023, the obstacle score for the per capita collection of public libraries (P5) showed an overall upward trend. Heilongjiang ranked among the top three provinces in all four years, with the degree of barriers gradually increasing, indicating that the region continues to face significant pressure in terms of book collection. Hubei also frequently appeared among the top three, but its obstacle level remained relatively stable. The obstacle level for the number of exhibitions held by public libraries (P6) increased, with Shanxi, Inner Mongolia, and Liaoning having higher obstacle levels in multiple years. The obstacle level for the number of various lectures organized by public libraries (P7) fluctuated across different years but remained relatively stable overall.

#### 3.3.2 Tobit regression analysis.

To further identify the factors influencing the degree of coupling coordination between PCSs and ED, we conduct a random effects Tobit regression analysis via Stata 17.0. In this study, the dependent variable, the degree of coupling coordination, ranged from 0 to 1 and exhibited a truncated distribution. Therefore, the Tobit model is used, with the truncation boundaries set at 0 and 1, representing the lower and upper bounds, respectively. The likelihood ratio (LR) test indicates that the random effects Tobit model provides a better fit. Accordingly, the regression analysis is performed using the random-effects Tobit model while controlling for year effects. The results are presented in Table 7.

**Table 7:** Tobit regression results.

Variables	Tobit random effects
P5	0.034***
	(3.272)
P6	0.013***
	(3.435)
P7	0.021***
	(5.431)
E1	0.001***
	(7.053)
E3	0.005***
	(8.336)
sigma_u	0.042***
	(6.906)
sigma_e	0.015***
	(23.279)
Constant	0.001
	(0.018)
Year	YES
Observations	310
Log likelihood	797.200
LR test of sigma_u = 0: chibar2(01)	416.46
Prob >= chibar2	0.000

Notes: The values in parentheses are z statistics; z = regression coefficient/standard error. ***, ** and * indicate significance at the 1%, 5% and 10% levels, respectively.

In total, regression coefficients for P5, P6, P7 and factors with values E1 and E3 were positive with t-value being significant at 1%, indicating an important effect to a different extent. In PCS system the regression coefficient of P5 is 0.034 which is the highest among all variables, indicating the significance of marginal impact of P5 on the degree of coupling coordination.This results reveal per capita collection of public libraries plays an important role in coordinated development of PCSs and economic growth. The regression coefficient P7 (0.021) is also of significance, second only to P5. This finding demonstrates that the number of different types of lectures hosted by public libraries has an important influence on the degree of coupling coordination. Conversely, the coefficient of P6 is 0.013. Although P6 is small in value, it is still important, which indicates that the number of exhibitions hosted by public libraries also has impact in terms of coordination. In the ED system, the coefficient E1 and E3 possess the value are 0.001 and 0.005 respectively, and they are both significant at 1%. These findings indicate that the total import and export value of goods and the market transaction volume of the technology industry exhibit a relatively stable and persistent impact on the increase in the coupling coordination degree. Though some variables’ coefficients are very small, their long-time stable growth suggest that things such as the economy’s fundamentals, technological innovation, and regions opening can continuously promote the interaction between PCSs and ED, thus supporting improvements in their overall coordination.

Additionally, as shown in Table 7, the model exhibits a satisfactory overall fit, with a log-likelihood value of 797.200, and passes the random effects LR test (chibar² = 416.46, p < 0.001), confirming the appropriateness of the Tobit model specification. sigma_u and sigma_e correspond to the variance of individual random effects and the variance of residuals within individuals, respectively. Their significance further indicates that both individual effects and random disturbances are nonnegligible in the model. Additionally, the LR test of sigma_u = 0: chibar2 (01) = 416.46, Prob >= chibar2 = 0.000, suggests the rejection of the null hypothesis sigma_u = 0, providing statistical justification for the inclusion of random effects in the model.

To address the potential issue of multicollinearity, this study calculates the variance inflation factor (VIF) for all the core independent variables, with the results presented in Table 8. Existing research indicates that a VIF value less than 5 suggests low multicollinearity, implying weak linear relationships among the independent variables and minimal impact on the stability of regression coefficient estimates.

**Table 8:** The variance inflation factor (VIF) for all the core variables.

Variables	VIF	1/VIF
P7	4.792	0.209
P6	4.419	0.226
E1	1.34	0.746
P5	1.277	0.783
E3	1.154	0.866
Mean_VIF	2.597	

As shown in Table 8, all the VIF values are below the empirical threshold of 5, with the highest value being 4.792 for variable P7 and the lowest being 1.154 for variable E3. The mean VIF (Mean_VIF) is 2.597, which is below the critical value of 5, indicating that the linear collinearity effect among the independent variables is weak and does not distort the coefficient estimates.

To verify the reliability of the research conclusions and avoid potential biases arising from model specification, variable selection, or data handling, this study conducts three types of robustness tests, as shown in Table 9.

**Table 9:** Robustness testing of three types of Tobit models.

Variables	Type (1)	Type (2)	Type (3)
P5	0.044***	0.048***	0.029**
	(3.772)	(3.394)	(2.402)
P6	0.013***	0.012***	0.013***
	(3.449)	(3.075)	(2.630)
P7	0.018***	0.018***	0.019***
	(4.556)	(4.368)	(4.176)
E5	0.024***		
	(7.873)		
E9	0.087***		
	(4.466)		
E1		0.001***	0.001***
		(4.385)	(4.846)
E3		0.004***	0.005***
		(6.688)	(5.246)
sigma_u	0.039***		0.043***
	(6.453)		(6.315)
sigma_e	0.015***		0.012***
	(23.061)		(18.507)
Constant	−0.887***	0.074**	0.032
	(−4.750)	(2.266)	(0.842)
Year	YES	YES	YES
Province	YES	YES	YES
Observations	310	310	217
Log likelihood	789.897	/	574.578
LR test of sigma u-0: chibar2(01)	398.85	/	306.42
Prob >= chibar2	0.000	/	0.000
R-squared	/	0.981	/

Notes: The values in parentheses are z statistics; z = regression coefficient/standard error. ***, ** and * indicate significance at the 1%, 5% and 10% levels, respectively.

Column (1) of Table 9 presents the test results when an alternative set of covariates is used. In the original model, variables E1 and E3 were used as the primary factors influencing the ED system. In this test, these indicators are replaced with alternative indicators E5 and E9 from the same system, and the model is re-estimated. The results show that the coefficients for the PCS system variables (P5, P6, and P7) remain significantly positive, which is consistent with the previous research direction. The alternative variables E5 and E9 are also theoretically expected and significant. The model’s log-likelihood is 789.897, and the likelihood ratio test p value is 0.000, confirming that the random effect specification remains appropriate. These results suggest that the core conclusions are not affected by the choice of covariate. Column (2) of Table 9 presents the test results with an alternative functional form. The original model uses a random-effects Tobit specification, and this test replaces it with a province-year fixed effects model to change the functional form of the model for robustness verification. The results show that the coefficients for the PCS system variables (P5, P6, and P7) remain significantly positive, with no substantive differences from the previous results. The model’s goodness of fit (R-squared) is high, and both the control variables Year and Province are statistically significant. This suggests that the core effects are not dependent on either random or fixed effects or on the specific functional form of the Tobit model. Column (3) of Table 9 presents the test results excluding potential imputed samples. Given that data from Tibet after 2020 have been imputed, this test excludes samples from after 2020 and re-estimates the model using only the original observations. The results show that the coefficients for the PCS system variables (P5, P6, and P7) remain significantly positive. Although the significance level for P5 decreases from 1% to 5%, it still meets the statistical significance requirement. The model’s likelihood ratio test p value is 0.000, and both sigma_u and sigma_e are significant. These findings indicate that the research conclusions are not affected by imputed data, confirming the robustness and reliability of the results.

## 4. Discussion

In this study, we analyze the intrinsic links between PCSs and ED in China. We seek to reveal how the two interact and synergistically evolve at different stages. We construct a PCS–ED evaluation index system, use a degree of coupled coordination model to assess the degree of coordination in each province, and use an obstacle degree model to analyze the factors that affect the coordinated development of the two systems. Accordingly, we identify the differences across various regions and highlight the problem of unbalanced development across regions.

Compared with the literature, the main contributions of this study are as follows. First, a more comprehensive PCS–ED evaluation index system covering multiple dimensions of PCSs and ED is constructed. Second, the coupling coordination degree model is used to evaluate the degree of PCS–ED coordination of 31 provinces and to analyze its spatiotemporal evolutionary characteristics. Third, the obstacle degree model is employed to analyze the factors that affect PCS–ED coordinated development and to identify the main obstacles.

The results of the comprehensive evaluation show that compared with PCSs, ED contributes more to the comprehensive evaluation, as ED is an important pillar of regional strength and plays a decisive role in a region’s economic foundation. However, the importance and scale of the PCS system are gradually increasing. A study of cultural participation in China’s major cities confirms this view, and the link between PCSs and urban development and growth in urban economies leads to an increase in residents’ income and promotes citizens’ participation in a wide range of cultural activities [45]. These findings differ from those of studies conducted in Western countries. Research from the United Kingdom indicates that cultural capital plays a significant role in promoting ED [2]. These results suggest that there may be differences in the relationship between cultural development and economic growth across different countries and regions. From a spatial distribution perspective, the comprehensive evaluation scores of provinces in the northern and southeastern regions are relatively high. These findings are the result of not only leading top-down policy guidance but also the efforts of local communities at the grassroots level. The western and northeastern provinces have relatively weak economic foundations, with economic growth rates and total outputs below the national average, which impacts their comprehensive evaluation scores. These regions have lagged in investments in and the development of PCSs, failing to effectively increase their comprehensive evaluation scores.

The results of the coupling coordination analysis reveal that China’s PCSs and EDs have advanced in terms of the degree of coupling coordination. However, there is still much room for improvement, and the goal is to move toward a high degree of coordination. The coupled and coordinated development of PCSs and EDs in China’s 31 provinces shows obvious regional differences and spatiotemporal evolutionary characteristics. The eastern coastal region, represented by Shanghai, Jiangsu, Zhejiang, Shandong and Guangdong, maintains a high degree of coupling coordination and forms a stable high-value agglomeration area. In contrast, the central provinces of Hunan and Jiangxi have made progress but are still at a low to medium level overall, which makes them transition zones between East and West China. The degree of coupling coordination in Northwest and Southwest China is generally low, with low or sub-low values. Empirical research findings indicate that PCS facilities and activities are scarce in Western China and that ecological thinking is lacking in the long-term process of constructing a PCS system [46]. This outcome is closely related to the current situation of a relatively limited ED scale and room for improvement in quality in these regions. This is consistent with findings from other developing countries. For example, research in India has shown that factors such as ED levels, government policies, and cultural infrastructure collectively influence cultural development [47]. Additionally, the PCS–ED coupling coordination degree in China’s 31 provinces clearly shows path dependence. Higher-level provinces are associated with a notable positive spatial link to lower-level provinces, while provinces at the same level also display greater stability. These findings suggest a significant positive spatial correlation.

The results of the obstacle analysis confirm that all 31 provinces in China face obstacles and challenges related to PCSs and ED to varying degrees. The concept of public culture encompasses the entire spectrum of media and popular culture, a concept that refers to the expression of public and personal politics as a contested domain using emotional communication [48]. In the PCS system, the per capita collection of public libraries, the number of exhibitions held by public libraries and the number of various lectures organized by public libraries are the main obstacles and are frequent and very high obstacles in most provinces. These findings indicate that libraries are generally deficient in the planning, organization and promotion of exhibitions and lectures. The services provided by libraries need to meet people’s needs for diverse and rich cultural activities [49]. In the ED system, the total import and export value of goods, the total retail sales of consumer goods, and the technology market transaction volume become obstacles in more than half of the provinces, suggesting that factors that affect ED exist in the goods trade, consumer markets, and technology markets. The proportion of the tertiary industry and the value added of the three major industries appear to be major obstacles in multiple provinces, and they reflect widespread issues in the development of the tertiary industry across provinces. These results are consistent with previous research conclusions, with some studies acknowledging the dominant position of the tertiary industry relative to other industries [50] and recognizing the importance of the tertiary industry [51].

This study has several limitations that should be considered. First, the research methods are limited. In this study, the degree of coupling coordination model and the obstacle degree model are used for empirical analysis. As these models are essentially descriptive, hypothesis testing and sensitivity analysis could not be conducted, which may limit the robustness of the results. In this study, ArcGIS and KDE are used for spatial visualization. Moreover, the spatial Markov chain is applied to explore spatial spillover effects. However, owing to objective constraints, a more comprehensive or advanced spatial econometric model is lacking. Second, the research data have several limitations. The data in this study are derived mainly from publicly available statistical yearbooks and reports, which may contain missing or inconsistent data and potentially affect the accuracy of the research results. Future studies may consider the use of more diverse data sources, such as survey data and interview data, to improve data quality and reliability. Third, the design of the indicator system has limitations. Although the PCS–ED evaluation indicator system constructed in this study covers multiple dimensions, there may still be some subjectivity in the selection of indicators or the setting of weights. In future research, more objective methods could be adopted, and more multidimensional indicators that reflect PCSs and ED could be incorporated to optimize the design of the indicator system.

## 5. Conclusion and policy suggestions

### 5.1 Conclusion

In this study, we analyze the coupled and coordinated development of PCSs and ED in 31 provinces in China from 2014 to 2023. The comprehensive evaluation reveals that ED contributes more than do PCSs do, but the importance and scale of PCSs have gradually increased over time. This study reveals that there are significant spatial differences in the degree of coupling and coordination between PCSs and ED, with relatively high degrees of coupling and coordination in the eastern coastal areas and relatively low degrees in the western and northeastern provinces. The degree of PCS–ED coupling coordination in China’s 31 provinces clearly shows path dependence. Higher-level provinces are associated with a notable positive spatial link to lower-level provinces, while provinces at the same level also display greater stability. These findings suggest a significant positive spatial correlation. In addition, we identify the main obstacles that affect the coupled and coordinated development of PCSs and ED, such as the inadequacy of libraries in terms of exhibitions and lectures and the impact of the import and export trade and technology transactions. The results of this study also indicate that there are both similarities and differences in the obstacles to ED across provinces. Notably, the indicator system may not comprehensively cover all relevant aspects, the research methods are not sufficiently comprehensive, and data are missing in some regions. Future research should aim to further improve the indicator system, collect more complete data, and conduct more extensive comparative analyses to provide a more scientific and comprehensive basis for policy formulation and regional development.

### 5.2 Policy suggestions

PCSs and ED are closely linked to the well-being of citizens and constitute important strategic priorities for national development. On the basis of the aforementioned findings, this paper identifies the key areas where lack of the integration of PCSs and ED and proposes corresponding policy recommendations.

Firstly, the PCS system should be optimized. PCSs represent a primary mean of protecting citizens’ fundamental cultural rights and serve as a vital mechanism for enhancing overall population quality and advancing the level of social civilization. The government should prioritize the development of public libraries, especially in less developed regions such as Heilongjiang and Hubei, where book collections remain insufficient. In these regions, fiscal transfers and some targeted programe should be implemented to encourage social donation and promote public-private partnership to expand library resources. Apart from expanding resources, it is important to improve the quality of library. Libraries should host regular exhibitions, conduct lectures, and organize small book clubs. They should also strive to transform the library itself into a space promotes both learning and community interaction, serving as a “third space” [52]. Furthermore, future investment should focus on expanding performing arts venues, increasing the utilization of existing facilities, promoting the establishment of community cultural centers, and supporting the creation of rural cultural stations. In less developed and remote rural regions, the cross-regional allocation of cultural resources should be encouraged. In eastern provinces where are well enrolled with cultural facilities and talent, should adopt paired assistance, resource sharing, and technical guidance to support the central and western regions. Mobile libraries, digital devices, and touring exhibitions can also be utilized to eliminate geographical obstacles and ensure that cultural services are inclusive and accessible through all regions.

Secondly, economic transformation and upgrading should be promoted. ED plays a key role in improving a nation’s aggregate strength and citizen’s quality of life. For China, it is imperative to shift focus from expansion and scale to quality and innovation. On one hand, promoting the quality of ED requires increase in support for technological innovation. Improving the quality of ED requires increased government investment in research and development (R&D), and offering incentives to encourage firms own internal R&D pursuits. In laggard provinces, establishing regional centers for scientific innovation and technology transfer can help translate scientific advancements into tangible products. On the other hand, the scale of ED should be expanded. It is important to encourage trade in goods, raise the total value of imports and exports, and optimize the trade structure. Particular emphasis should be placed on increasing the technical sophistication and add values of exports, especially in central and western provinces with outdated industrial structures. Active participation in international labor markets and regional trade agreements can promote economic upgrading that benefit less developed provinces, rather than focusing on coastal provinces.

Thirdly, we should strengthen regional coordination and development. Region development imbalance remians the most significant structural issue in China. On the contrary, it is necessary to increase the support of PCSs in central and western regions. Addressing this issue requires increased public investment in cultural infrastructure, targeted subsidies for grassroots cultural stations, and training programs to increase the capacity of local cultural workers. To promote cross-regional allocation of cross-resources, eastern regions with a surplus of both cultural resources and expertise should increase their efforts in assistance programs, cultural resources sharing, training, and administrative support for the centeral and western regions; collaborations such as shared exhibitions, touring events, digital cultural platforms, etc., can enable high-quality cultural services to reach underdeveloped regions. Coordinated regional ED should be promoted on the other hand. Overcoming administrative barriers, and facilitaing flexible flows of capital, labour and knowledge will allow regions to to complement one another through their respective economic advantages. Provinces facing development challenges should adopt differentiated strategies. Resource-based provinces could go for a green transfomation, while agrarian provinces may focus on modern agribusiness linked with cultural tourism. Regional development planning should be guided by local resource endowments and development potential. This approach can realize a growth model with multi-level, differential, and synchronous characteristics, to narrow regional disparities on the basis of sustainable high-quality national development.

Accordingly, this study of the coupling relationship between the PCS and ED advances the application of coupling coordination theory in the field of regional development and contributes to understanding the theoretical connotations of regional development. This study also has important theoretical value in terms of clarifying the relationship between PCSs and ED as well as the feedback mechanism of the effect of ED on PCSs, laying a solid theoretical foundation for subsequent research. The results provide a scientific basis for government departments to formulate policies and guide the rational allocation of resources. In this way, differentiated policies can be formulated for the coupled and coordinated development levels of different regions to achieve synergy between PCSs and ED.

## Supporting information

S1 File The calculation process of the comprehensive evaluation value of indicators P1 and E1. (DOCX)

S1 Fig A sensitivity plot for the neighboring bandwidth at the national level for 2019. (TIF)
