# Supplementary material for: The coupling coordination relationship and obstacle factors of public cultural services and economic development: A case study of 31 regions in China
Source: PLoS One. 2026 Jan 20;21(1):e0341305. doi: 10.1371/journal.pone.0341305 (PMC12818661; doi:10.1371/journal.pone.0341305)
Supplement: S1 File — (DOCX) [file pone.0341305.s001.docx]

## Comprehensive development level evaluation model

The PCS (corresponding to the system where indicator P1 is located) and ED (corresponding to the system where indicator E1 is located) systems are independent of each other while having interactive effects. In their comprehensive evaluation model, P1 (a representative indicator of the PCS system) and E1 (a representative indicator of the ED system) serve as core secondary indicators, and objective weighting is required to determine their importance. Studies have shown that the entropy weight method can effectively measure the differences among indicators and convert these differences as well as the importance of indicators into quantified results, thereby providing a scientific basis for comprehensive evaluation.

**Step 1: Data Standardization**

Due to differences in measurement units and value scales between indicators E1 and P1, data standardization is a necessary prerequisite to eliminate the interference of these differences on the analysis results.

In this study, the extreme value method is adopted for data standardization, and the formula is as follows (Equation 1). The results are shown in columns (1) and (2) of Table 1.

| $Y_{ij}=\frac{y_{ij}-min(y_{ij})}{max(y_{ij})-min(y_{ij})}+0.01$ | (1) |
| --- | --- |

Equation (1) is used to standardize the data through the extreme value method. In this equation, *Y_ij_* represents the data of the *j*-th indicator in the *i*-th year after the standardization process, *y_ij_* is the original data of the *j*-th indicator in the *i*-th year, and *max(y_ij_)* and *min(y_ij_)* identify the maximum and minimum values of the *j*-th indicator in the *i*-th year, respectively. If there are zero values in the original data of E1 or P1, 0.01 is uniformly added for processing with reference to relevant literature, so as to avoid the invalidation of subsequent weighting results.

**Step 2: Calculation of Indicator Information Entropy**

Based on the standardized data, the information entropy of E1 and P1 is calculated respectively to reflect the degree of dispersion of the indicator data. The calculation formula is as follows (Equation 2). The results are shown in columns (3) and (4) of Table 1.

| $H_{j}=-(\frac{1}{\ln n})\cdot\sum_{i=1}^{n} c_{j}\ln c_{j}$ | (2) |
| --- | --- |

Equation (2) is used to calculate the entropy value of a secondary indicator. In this equation, *H_j_* represents the information entropy of the *j*-th indicator. *n* is the number of samples; *c_j_* is the proportion of the *j*-th indicator in the *j*-th sample; and *ln* is the natural logarithm, which is used to convert probability into a measure of information quantity.

**Step 3: Calculation of Indicator Weights**

The information utility value reflects the contribution capacity of an indicator to the comprehensive evaluation. A higher utility value indicates greater importance of the indicator in the evaluation. The calculation formula is as follows (Equation 3).

| $\varphi_{j}=\frac{d_{j}}{\sum_{j=1}^{m} d_{j}}$ *;*$d_{j}=1-H_{j}$ | (3) |
| --- | --- |

Equation (3) is used to calculate the weight of a secondary indicator in this system. When the information utility value is higher, the importance of the indicator is greater in the overall evaluation. In this equation, *φ_j_* represents the information utility value of the *j*-th indicator, and *m* is the total number of indicators. *d_j_* represents the difference coefficient of the *j*-th indicator, and *H_j_* is the information entropy of the *j*-th indicator.

The weighting method is used to calculate the final weights of E1 and P1, so as to clarify the degree of influence of the two indicators on the comprehensive evaluation results [32]. The calculation formula is as follows (Equation 4). The results are shown in columns (5) and (6) of Table 1.

| $W_{n}=\sum_{j=1}^{m} \varphi_{ij}Y_{ij}$ | (4) |
| --- | --- |

Equation (4) is used to calculate the weight of each evaluation indicator by the weighting method, which is denoted as *W_i_* (*i*=1, 2..., k), where *k* indicates the number of indicators. When the weight of an indicator is higher, its impact on the final evaluation result is greater, indicating that it is more important.

**Step 4: Calculation of Comprehensive Evaluation Level**

The standardized data of E1 and P1 are integrated into a comprehensive evaluation value through weighted averaging to reflect the overall level of the systems represented by the two indicators. The calculation formula is as follows (Equation 5). The results are shown in columns (7) and (8) of Table 1.

| $U_{n}=\sum_{i=1}^{m} W_{ij}Y_{ij}$ *,* $n=1,2$ *,*$\sum_{i=1}^{m} W_{ij}=1$ | (5) |
| --- | --- |

Equation (5) is the comprehensive evaluation value of the indicators, which is obtained by combining multiple indicators into a comprehensive score through weighted averaging. *U_n_* represents the comprehensive evaluation value of the *n*-th object, which is obtained by multiplying the standardized values (*Y_ij_*) of each indicator by the corresponding weights (*W_ij_*) and then summing them. *U_P1_* and *U_E1_* are the combined evaluation values of P1 and E1, respectively.

**Table 1. Calculation result of comprehensive score of indicators.**

| **Provinces** | **Year** | **(1)** | **(2)** | **(3)** | **(4)** | **(5)** | **(6)** | **(7)** | **(8)** |
| --- | --- | --- | --- | --- | --- | --- | --- | --- | --- |
|  |  | **Data standardization** | | **Indicator information entropy** | | **Indicator weight matrix** | | **Comprehensive score of indicators** | |
|  |  | **P1** | **E1** | **P1** | **E1** | **P1** | **E1** | **U_P1_** | **U_E1_** |
| Beijing | 2014 | 0.0560 | 0.3246 | 0.0561 | 0.3247 | 0.0008 | 0.0087 | 0.0060 | 0.0768 |
| Beijing | 2015 | 0.0545 | 0.2495 | 0.0546 | 0.2496 | 0.0008 | 0.0067 | 0.0058 | 0.0590 |
| Beijing | 2016 | 0.0560 | 0.2205 | 0.0561 | 0.2206 | 0.0008 | 0.0059 | 0.0060 | 0.0522 |
| Beijing | 2017 | 0.1014 | 0.2528 | 0.1015 | 0.2529 | 0.0014 | 0.0068 | 0.0108 | 0.0598 |
| Beijing | 2018 | 0.1180 | 0.3222 | 0.1181 | 0.3223 | 0.0017 | 0.0086 | 0.0125 | 0.0762 |
| Beijing | 2019 | 0.1165 | 0.3253 | 0.1166 | 0.3254 | 0.0016 | 0.0087 | 0.0124 | 0.0770 |
| Beijing | 2020 | 0.1150 | 0.2628 | 0.1151 | 0.2629 | 0.0016 | 0.0071 | 0.0122 | 0.0622 |
| Beijing | 2021 | 0.1135 | 0.3680 | 0.1136 | 0.3681 | 0.0016 | 0.0099 | 0.0121 | 0.0871 |
| Beijing | 2022 | 0.1180 | 0.4266 | 0.1181 | 0.4267 | 0.0017 | 0.0114 | 0.0125 | 0.1009 |
| Beijing | 2023 | 0.0983 | 0.4051 | 0.0984 | 0.4052 | 0.0014 | 0.0109 | 0.0105 | 0.0958 |
| Tianjin | 2014 | 0.0272 | 0.1044 | 0.0273 | 0.1045 | 0.0004 | 0.0028 | 0.0029 | 0.0247 |
| Tianjin | 2015 | 0.0272 | 0.0891 | 0.0273 | 0.0892 | 0.0004 | 0.0024 | 0.0029 | 0.0211 |
| Tianjin | 2016 | 0.0272 | 0.0800 | 0.0273 | 0.0801 | 0.0004 | 0.0022 | 0.0029 | 0.0190 |
| Tianjin | 2017 | 0.0878 | 0.0881 | 0.0879 | 0.0882 | 0.0012 | 0.0024 | 0.0093 | 0.0209 |
| Tianjin | 2018 | 0.0923 | 0.0956 | 0.0924 | 0.0957 | 0.0013 | 0.0026 | 0.0098 | 0.0226 |
| Tianjin | 2019 | 0.0968 | 0.0831 | 0.0969 | 0.0832 | 0.0014 | 0.0022 | 0.0103 | 0.0197 |
| Tianjin | 2020 | 0.1014 | 0.0829 | 0.1015 | 0.0830 | 0.0014 | 0.0022 | 0.0108 | 0.0196 |
| Tianjin | 2021 | 0.0983 | 0.1034 | 0.0984 | 0.1035 | 0.0014 | 0.0028 | 0.0105 | 0.0245 |
| Tianjin | 2022 | 0.1029 | 0.0972 | 0.1030 | 0.0973 | 0.0015 | 0.0026 | 0.0109 | 0.0230 |
| Tianjin | 2023 | 0.0741 | 0.0888 | 0.0742 | 0.0889 | 0.0010 | 0.0024 | 0.0079 | 0.0210 |
| Hebei | 2014 | 0.1528 | 0.0466 | 0.1529 | 0.0467 | 0.0022 | 0.0013 | 0.0162 | 0.0110 |
| Hebei | 2015 | 0.1558 | 0.0400 | 0.1559 | 0.0401 | 0.0022 | 0.0011 | 0.0166 | 0.0095 |
| Hebei | 2016 | 0.1619 | 0.0362 | 0.1620 | 0.0363 | 0.0023 | 0.0010 | 0.0172 | 0.0086 |
| Hebei | 2017 | 0.1785 | 0.0387 | 0.1786 | 0.0388 | 0.0025 | 0.0010 | 0.0190 | 0.0092 |
| Hebei | 2018 | 0.1967 | 0.0419 | 0.1968 | 0.0420 | 0.0028 | 0.0011 | 0.0209 | 0.0099 |
| Hebei | 2019 | 0.1997 | 0.0451 | 0.1998 | 0.0452 | 0.0028 | 0.0012 | 0.0212 | 0.0107 |
| Hebei | 2020 | 0.2179 | 0.0502 | 0.2180 | 0.0503 | 0.0031 | 0.0014 | 0.0231 | 0.0119 |
| Hebei | 2021 | 0.2542 | 0.0653 | 0.2543 | 0.0654 | 0.0036 | 0.0018 | 0.0270 | 0.0155 |
| Hebei | 2022 | 0.2738 | 0.0642 | 0.2739 | 0.0643 | 0.0039 | 0.0017 | 0.0291 | 0.0152 |
| Hebei | 2023 | 0.2163 | 0.0645 | 0.2164 | 0.0646 | 0.0031 | 0.0017 | 0.0230 | 0.0153 |
| Shanxi | 2014 | 0.1498 | 0.0125 | 0.1499 | 0.0126 | 0.0021 | 0.0003 | 0.0159 | 0.0030 |
| Shanxi | 2015 | 0.1437 | 0.0112 | 0.1438 | 0.0113 | 0.0020 | 0.0003 | 0.0153 | 0.0027 |
| Shanxi | 2016 | 0.1437 | 0.0128 | 0.1438 | 0.0129 | 0.0020 | 0.0004 | 0.0153 | 0.0031 |
| Shanxi | 2017 | 0.1422 | 0.0132 | 0.1423 | 0.0133 | 0.0020 | 0.0004 | 0.0151 | 0.0031 |
| Shanxi | 2018 | 0.1452 | 0.0160 | 0.1453 | 0.0161 | 0.0021 | 0.0004 | 0.0154 | 0.0038 |
| Shanxi | 2019 | 0.1422 | 0.0162 | 0.1423 | 0.0163 | 0.0020 | 0.0004 | 0.0151 | 0.0039 |
| Shanxi | 2020 | 0.1558 | 0.0168 | 0.1559 | 0.0169 | 0.0022 | 0.0005 | 0.0166 | 0.0040 |
| Shanxi | 2021 | 0.1694 | 0.0267 | 0.1695 | 0.0268 | 0.0024 | 0.0007 | 0.0180 | 0.0064 |
| Shanxi | 2022 | 0.1694 | 0.0213 | 0.1695 | 0.0214 | 0.0024 | 0.0006 | 0.0180 | 0.0051 |
| Shanxi | 2023 | 0.1225 | 0.0185 | 0.1226 | 0.0186 | 0.0017 | 0.0005 | 0.0130 | 0.0044 |
| Inner Mongolia | 2014 | 0.4493 | 0.0111 | 0.4494 | 0.0112 | 0.0063 | 0.0003 | 0.0477 | 0.0027 |
| Inner Mongolia | 2015 | 0.4660 | 0.0097 | 0.4661 | 0.0098 | 0.0066 | 0.0003 | 0.0495 | 0.0023 |
| Inner Mongolia | 2016 | 0.4735 | 0.0089 | 0.4736 | 0.0090 | 0.0067 | 0.0002 | 0.0503 | 0.0021 |
| Inner Mongolia | 2017 | 0.4811 | 0.0106 | 0.4812 | 0.0107 | 0.0068 | 0.0003 | 0.0511 | 0.0025 |
| Inner Mongolia | 2018 | 0.4917 | 0.0120 | 0.4918 | 0.0121 | 0.0069 | 0.0003 | 0.0522 | 0.0029 |
| Inner Mongolia | 2019 | 0.5159 | 0.0122 | 0.5160 | 0.0123 | 0.0073 | 0.0003 | 0.0548 | 0.0029 |
| Inner Mongolia | 2020 | 0.5492 | 0.0117 | 0.5493 | 0.0118 | 0.0077 | 0.0003 | 0.0583 | 0.0028 |
| Inner Mongolia | 2021 | 0.5477 | 0.0147 | 0.5478 | 0.0148 | 0.0077 | 0.0004 | 0.0582 | 0.0035 |
| Inner Mongolia | 2022 | 0.5583 | 0.0174 | 0.5584 | 0.0175 | 0.0079 | 0.0005 | 0.0593 | 0.0041 |
| Inner Mongolia | 2023 | 0.4932 | 0.0215 | 0.4933 | 0.0216 | 0.0069 | 0.0006 | 0.0524 | 0.0051 |
| Liaoning | 2014 | 0.2769 | 0.0889 | 0.2770 | 0.0890 | 0.0039 | 0.0024 | 0.0294 | 0.0210 |
| Liaoning | 2015 | 0.3328 | 0.0748 | 0.3329 | 0.0749 | 0.0047 | 0.0020 | 0.0354 | 0.0177 |
| Liaoning | 2016 | 0.4100 | 0.0674 | 0.4101 | 0.0675 | 0.0058 | 0.0018 | 0.0435 | 0.0160 |
| Liaoning | 2017 | 0.4599 | 0.0775 | 0.4600 | 0.0776 | 0.0065 | 0.0021 | 0.0488 | 0.0184 |
| Liaoning | 2018 | 0.5038 | 0.0893 | 0.5039 | 0.0894 | 0.0071 | 0.0024 | 0.0535 | 0.0212 |
| Liaoning | 2019 | 0.5477 | 0.0821 | 0.5478 | 0.0822 | 0.0077 | 0.0022 | 0.0582 | 0.0194 |
| Liaoning | 2020 | 0.6082 | 0.0739 | 0.6083 | 0.0740 | 0.0086 | 0.0020 | 0.0646 | 0.0175 |
| Liaoning | 2021 | 0.6369 | 0.0932 | 0.6370 | 0.0933 | 0.0090 | 0.0025 | 0.0676 | 0.0221 |
| Liaoning | 2022 | 0.6475 | 0.0925 | 0.6476 | 0.0926 | 0.0091 | 0.0025 | 0.0688 | 0.0219 |
| Liaoning | 2023 | 0.3056 | 0.0850 | 0.3057 | 0.0851 | 0.0043 | 0.0023 | 0.0325 | 0.0201 |
| Jilin | 2014 | 0.1422 | 0.0204 | 0.1423 | 0.0205 | 0.0020 | 0.0006 | 0.0151 | 0.0048 |
| Jilin | 2015 | 0.1422 | 0.0145 | 0.1423 | 0.0146 | 0.0020 | 0.0004 | 0.0151 | 0.0035 |
| Jilin | 2016 | 0.1422 | 0.0142 | 0.1423 | 0.0143 | 0.0020 | 0.0004 | 0.0151 | 0.0034 |
| Jilin | 2017 | 0.1800 | 0.0143 | 0.1801 | 0.0144 | 0.0025 | 0.0004 | 0.0191 | 0.0034 |
| Jilin | 2018 | 0.1876 | 0.0159 | 0.1877 | 0.0160 | 0.0026 | 0.0004 | 0.0199 | 0.0038 |
| Jilin | 2019 | 0.1906 | 0.0145 | 0.1907 | 0.0146 | 0.0027 | 0.0004 | 0.0203 | 0.0035 |
| Jilin | 2020 | 0.1937 | 0.0142 | 0.1938 | 0.0143 | 0.0027 | 0.0004 | 0.0206 | 0.0034 |
| Jilin | 2021 | 0.2058 | 0.0179 | 0.2059 | 0.0180 | 0.0029 | 0.0005 | 0.0219 | 0.0043 |
| Jilin | 2022 | 0.2058 | 0.0180 | 0.2059 | 0.0181 | 0.0029 | 0.0005 | 0.0219 | 0.0043 |
| Jilin | 2023 | 0.1483 | 0.0184 | 0.1484 | 0.0185 | 0.0021 | 0.0005 | 0.0158 | 0.0044 |
| Heilongjiang | 2014 | 0.3616 | 0.0302 | 0.3617 | 0.0303 | 0.0051 | 0.0008 | 0.0384 | 0.0072 |
| Heilongjiang | 2015 | 0.4660 | 0.0162 | 0.4661 | 0.0163 | 0.0066 | 0.0004 | 0.0495 | 0.0039 |
| Heilongjiang | 2016 | 0.5885 | 0.0127 | 0.5886 | 0.0128 | 0.0083 | 0.0003 | 0.0625 | 0.0030 |
| Heilongjiang | 2017 | 0.7277 | 0.0145 | 0.7278 | 0.0146 | 0.0103 | 0.0004 | 0.0773 | 0.0034 |
| Heilongjiang | 2018 | 0.7761 | 0.0204 | 0.7762 | 0.0205 | 0.0109 | 0.0006 | 0.0824 | 0.0049 |
| Heilongjiang | 2019 | 0.8124 | 0.0210 | 0.8125 | 0.0211 | 0.0114 | 0.0006 | 0.0863 | 0.0050 |
| Heilongjiang | 2020 | 0.8669 | 0.0171 | 0.8670 | 0.0172 | 0.0122 | 0.0005 | 0.0921 | 0.0041 |
| Heilongjiang | 2021 | 0.9455 | 0.0239 | 0.9456 | 0.0240 | 0.0133 | 0.0006 | 0.1004 | 0.0057 |
| Heilongjiang | 2022 | 1.0000 | 0.0308 | 1.0001 | 0.0309 | 0.0141 | 0.0008 | 0.1062 | 0.0073 |
| Heilongjiang | 2023 | 0.5204 | 0.0329 | 0.5205 | 0.0330 | 0.0073 | 0.0009 | 0.0553 | 0.0078 |
| Shanghai | 2014 | 0.2602 | 0.3644 | 0.2603 | 0.3645 | 0.0037 | 0.0098 | 0.0276 | 0.0862 |
| Shanghai | 2015 | 0.2617 | 0.3509 | 0.2618 | 0.3510 | 0.0037 | 0.0094 | 0.0278 | 0.0830 |
| Shanghai | 2016 | 0.2617 | 0.3388 | 0.2618 | 0.3389 | 0.0037 | 0.0091 | 0.0278 | 0.0802 |
| Shanghai | 2017 | 0.2723 | 0.3720 | 0.2724 | 0.3721 | 0.0038 | 0.0100 | 0.0289 | 0.0880 |
| Shanghai | 2018 | 0.2723 | 0.4029 | 0.2724 | 0.4030 | 0.0038 | 0.0108 | 0.0289 | 0.0953 |
| Shanghai | 2019 | 0.3586 | 0.3859 | 0.3587 | 0.3860 | 0.0051 | 0.0103 | 0.0381 | 0.0913 |
| Shanghai | 2020 | 0.4418 | 0.3936 | 0.4419 | 0.3937 | 0.0062 | 0.0106 | 0.0469 | 0.0931 |
| Shanghai | 2021 | 0.5068 | 0.4130 | 0.5069 | 0.4131 | 0.0071 | 0.0111 | 0.0538 | 0.0977 |
| Shanghai | 2022 | 0.5083 | 0.4342 | 0.5084 | 0.4343 | 0.0072 | 0.0116 | 0.0540 | 0.1027 |
| Shanghai | 2023 | 0.3313 | 0.4682 | 0.3314 | 0.4683 | 0.0047 | 0.0126 | 0.0352 | 0.1108 |
| Jiangsu | 2014 | 0.0212 | 0.4403 | 0.0213 | 0.4404 | 0.0003 | 0.0118 | 0.0023 | 0.1042 |
| Jiangsu | 2015 | 0.0212 | 0.4262 | 0.0213 | 0.4263 | 0.0003 | 0.0114 | 0.0023 | 0.1008 |
| Jiangsu | 2016 | 0.0212 | 0.3979 | 0.0213 | 0.3980 | 0.0003 | 0.0107 | 0.0023 | 0.0941 |
| Jiangsu | 2017 | 0.0227 | 0.4618 | 0.0228 | 0.4619 | 0.0003 | 0.0124 | 0.0024 | 0.1093 |
| Jiangsu | 2018 | 0.0227 | 0.5188 | 0.0228 | 0.5189 | 0.0003 | 0.0139 | 0.0024 | 0.1227 |
| Jiangsu | 2019 | 0.0348 | 0.4919 | 0.0349 | 0.4920 | 0.0005 | 0.0132 | 0.0037 | 0.1164 |
| Jiangsu | 2020 | 0.0469 | 0.5023 | 0.0470 | 0.5024 | 0.0007 | 0.0135 | 0.0050 | 0.1188 |
| Jiangsu | 2021 | 0.0530 | 0.5523 | 0.0531 | 0.5524 | 0.0008 | 0.0148 | 0.0056 | 0.1307 |
| Jiangsu | 2022 | 0.0605 | 0.5581 | 0.0606 | 0.5582 | 0.0009 | 0.0150 | 0.0064 | 0.1320 |
| Jiangsu | 2023 | 0.0439 | 0.5830 | 0.0440 | 0.5831 | 0.0006 | 0.0156 | 0.0047 | 0.1379 |
| Zhejiang | 2014 | 0.0893 | 0.2773 | 0.0894 | 0.2774 | 0.0013 | 0.0074 | 0.0095 | 0.0656 |
| Zhejiang | 2015 | 0.0908 | 0.2708 | 0.0909 | 0.2709 | 0.0013 | 0.0073 | 0.0097 | 0.0641 |
| Zhejiang | 2016 | 0.0923 | 0.2629 | 0.0924 | 0.2630 | 0.0013 | 0.0071 | 0.0098 | 0.0622 |
| Zhejiang | 2017 | 0.0923 | 0.2952 | 0.0924 | 0.2953 | 0.0013 | 0.0079 | 0.0098 | 0.0698 |
| Zhejiang | 2018 | 0.0923 | 0.3377 | 0.0924 | 0.3378 | 0.0013 | 0.0091 | 0.0098 | 0.0799 |
| Zhejiang | 2019 | 0.0923 | 0.3494 | 0.0924 | 0.3495 | 0.0013 | 0.0094 | 0.0098 | 0.0827 |
| Zhejiang | 2020 | 0.0923 | 0.3817 | 0.0924 | 0.3818 | 0.0013 | 0.0102 | 0.0098 | 0.0903 |
| Zhejiang | 2021 | 0.0923 | 0.5009 | 0.0924 | 0.5010 | 0.0013 | 0.0134 | 0.0098 | 0.1185 |
| Zhejiang | 2022 | 0.0923 | 0.5495 | 0.0924 | 0.5496 | 0.0013 | 0.0147 | 0.0098 | 0.1300 |
| Zhejiang | 2023 | 0.0938 | 0.5444 | 0.0939 | 0.5445 | 0.0013 | 0.0146 | 0.0100 | 0.1288 |
| Anhui | 2014 | 0.1120 | 0.0382 | 0.1121 | 0.0383 | 0.0016 | 0.0010 | 0.0119 | 0.0091 |
| Anhui | 2015 | 0.1089 | 0.0372 | 0.1090 | 0.0373 | 0.0015 | 0.0010 | 0.0116 | 0.0088 |
| Anhui | 2016 | 0.1104 | 0.0345 | 0.1105 | 0.0346 | 0.0016 | 0.0009 | 0.0117 | 0.0082 |
| Anhui | 2017 | 0.1558 | 0.0417 | 0.1559 | 0.0418 | 0.0022 | 0.0011 | 0.0166 | 0.0099 |
| Anhui | 2018 | 0.1558 | 0.0489 | 0.1559 | 0.0490 | 0.0022 | 0.0013 | 0.0166 | 0.0116 |
| Anhui | 2019 | 0.1558 | 0.0535 | 0.1559 | 0.0536 | 0.0022 | 0.0014 | 0.0166 | 0.0127 |
| Anhui | 2020 | 0.1558 | 0.0613 | 0.1559 | 0.0614 | 0.0022 | 0.0016 | 0.0166 | 0.0145 |
| Anhui | 2021 | 0.1528 | 0.0835 | 0.1529 | 0.0836 | 0.0022 | 0.0022 | 0.0162 | 0.0198 |
| Anhui | 2022 | 0.1528 | 0.0882 | 0.1529 | 0.0883 | 0.0022 | 0.0024 | 0.0162 | 0.0209 |
| Anhui | 2023 | 0.1362 | 0.0892 | 0.1363 | 0.0893 | 0.0019 | 0.0024 | 0.0145 | 0.0211 |
| Fujian | 2014 | 0.2330 | 0.1384 | 0.2331 | 0.1385 | 0.0033 | 0.0037 | 0.0248 | 0.0328 |
| Fujian | 2015 | 0.2330 | 0.1318 | 0.2331 | 0.1319 | 0.0033 | 0.0035 | 0.0248 | 0.0312 |
| Fujian | 2016 | 0.2602 | 0.1224 | 0.2603 | 0.1225 | 0.0037 | 0.0033 | 0.0276 | 0.0290 |
| Fujian | 2017 | 0.2708 | 0.1335 | 0.2709 | 0.1336 | 0.0038 | 0.0036 | 0.0288 | 0.0316 |
| Fujian | 2018 | 0.2829 | 0.1463 | 0.2830 | 0.1464 | 0.0040 | 0.0039 | 0.0301 | 0.0346 |
| Fujian | 2019 | 0.2859 | 0.1507 | 0.2860 | 0.1508 | 0.0040 | 0.0040 | 0.0304 | 0.0357 |
| Fujian | 2020 | 0.2829 | 0.1589 | 0.2830 | 0.1590 | 0.0040 | 0.0043 | 0.0301 | 0.0376 |
| Fujian | 2021 | 0.2617 | 0.2229 | 0.2618 | 0.2230 | 0.0037 | 0.0060 | 0.0278 | 0.0528 |
| Fujian | 2022 | 0.2617 | 0.2322 | 0.2618 | 0.2323 | 0.0037 | 0.0062 | 0.0278 | 0.0550 |
| Fujian | 2023 | 0.1937 | 0.2192 | 0.1938 | 0.2193 | 0.0027 | 0.0059 | 0.0206 | 0.0519 |
| Jiangxi | 2014 | 0.1437 | 0.0332 | 0.1438 | 0.0333 | 0.0020 | 0.0009 | 0.0153 | 0.0079 |
| Jiangxi | 2015 | 0.1452 | 0.0329 | 0.1453 | 0.0330 | 0.0021 | 0.0009 | 0.0154 | 0.0078 |
| Jiangxi | 2016 | 0.1528 | 0.0311 | 0.1529 | 0.0312 | 0.0022 | 0.0008 | 0.0162 | 0.0074 |
| Jiangxi | 2017 | 0.2027 | 0.0345 | 0.2028 | 0.0346 | 0.0029 | 0.0009 | 0.0215 | 0.0082 |
| Jiangxi | 2018 | 0.2239 | 0.0374 | 0.2240 | 0.0375 | 0.0032 | 0.0010 | 0.0238 | 0.0089 |
| Jiangxi | 2019 | 0.2330 | 0.0395 | 0.2331 | 0.0396 | 0.0033 | 0.0011 | 0.0248 | 0.0094 |
| Jiangxi | 2020 | 0.2345 | 0.0451 | 0.2346 | 0.0452 | 0.0033 | 0.0012 | 0.0249 | 0.0107 |
| Jiangxi | 2021 | 0.2693 | 0.0600 | 0.2694 | 0.0601 | 0.0038 | 0.0016 | 0.0286 | 0.0142 |
| Jiangxi | 2022 | 0.2602 | 0.0774 | 0.2603 | 0.0775 | 0.0037 | 0.0021 | 0.0276 | 0.0183 |
| Jiangxi | 2023 | 0.2284 | 0.0631 | 0.2285 | 0.0632 | 0.0032 | 0.0017 | 0.0243 | 0.0150 |
| Shandong | 2014 | 0.2421 | 0.2162 | 0.2422 | 0.2163 | 0.0034 | 0.0058 | 0.0257 | 0.0512 |
| Shandong | 2015 | 0.2527 | 0.1878 | 0.2528 | 0.1879 | 0.0036 | 0.0050 | 0.0268 | 0.0445 |
| Shandong | 2016 | 0.2527 | 0.1830 | 0.2528 | 0.1831 | 0.0036 | 0.0049 | 0.0268 | 0.0433 |
| Shandong | 2017 | 0.2905 | 0.2054 | 0.2906 | 0.2055 | 0.0041 | 0.0055 | 0.0309 | 0.0486 |
| Shandong | 2018 | 0.2980 | 0.2283 | 0.2981 | 0.2284 | 0.0042 | 0.0061 | 0.0317 | 0.0540 |
| Shandong | 2019 | 0.3253 | 0.2319 | 0.3254 | 0.2320 | 0.0046 | 0.0062 | 0.0346 | 0.0549 |
| Shandong | 2020 | 0.3419 | 0.2501 | 0.3420 | 0.2502 | 0.0048 | 0.0067 | 0.0363 | 0.0592 |
| Shandong | 2021 | 0.3313 | 0.3544 | 0.3314 | 0.3545 | 0.0047 | 0.0095 | 0.0352 | 0.0838 |
| Shandong | 2022 | 0.3343 | 0.3772 | 0.3344 | 0.3773 | 0.0047 | 0.0101 | 0.0355 | 0.0893 |
| Shandong | 2023 | 0.2678 | 0.3628 | 0.2679 | 0.3629 | 0.0038 | 0.0097 | 0.0284 | 0.0858 |
| Henan | 2014 | 0.2012 | 0.0506 | 0.2013 | 0.0507 | 0.0028 | 0.0014 | 0.0214 | 0.0120 |
| Henan | 2015 | 0.2012 | 0.0574 | 0.2013 | 0.0575 | 0.0028 | 0.0015 | 0.0214 | 0.0136 |
| Henan | 2016 | 0.2027 | 0.0554 | 0.2028 | 0.0555 | 0.0029 | 0.0015 | 0.0215 | 0.0131 |
| Henan | 2017 | 0.2042 | 0.0604 | 0.2043 | 0.0605 | 0.0029 | 0.0016 | 0.0217 | 0.0143 |
| Henan | 2018 | 0.2118 | 0.0645 | 0.2119 | 0.0646 | 0.0030 | 0.0017 | 0.0225 | 0.0153 |
| Henan | 2019 | 0.2103 | 0.0643 | 0.2104 | 0.0644 | 0.0030 | 0.0017 | 0.0223 | 0.0152 |
| Henan | 2020 | 0.2542 | 0.0758 | 0.2543 | 0.0759 | 0.0036 | 0.0020 | 0.0270 | 0.0180 |
| Henan | 2021 | 0.2799 | 0.0991 | 0.2800 | 0.0992 | 0.0039 | 0.0027 | 0.0297 | 0.0235 |
| Henan | 2022 | 0.3011 | 0.0991 | 0.3012 | 0.0992 | 0.0042 | 0.0027 | 0.0320 | 0.0235 |
| Henan | 2023 | 0.2511 | 0.0898 | 0.2512 | 0.0899 | 0.0035 | 0.0024 | 0.0267 | 0.0213 |
| Hubei | 2014 | 0.3691 | 0.0334 | 0.3692 | 0.0335 | 0.0052 | 0.0009 | 0.0392 | 0.0079 |
| Hubei | 2015 | 0.3691 | 0.0354 | 0.3692 | 0.0355 | 0.0052 | 0.0010 | 0.0392 | 0.0084 |
| Hubei | 2016 | 0.4024 | 0.0306 | 0.4025 | 0.0307 | 0.0057 | 0.0008 | 0.0427 | 0.0073 |
| Hubei | 2017 | 0.4992 | 0.0360 | 0.4993 | 0.0361 | 0.0070 | 0.0010 | 0.0530 | 0.0085 |
| Hubei | 2018 | 0.4992 | 0.0410 | 0.4993 | 0.0411 | 0.0070 | 0.0011 | 0.0530 | 0.0097 |
| Hubei | 2019 | 0.5083 | 0.0444 | 0.5084 | 0.0445 | 0.0072 | 0.0012 | 0.0540 | 0.0105 |
| Hubei | 2020 | 0.5023 | 0.0484 | 0.5024 | 0.0485 | 0.0071 | 0.0013 | 0.0533 | 0.0115 |
| Hubei | 2021 | 0.5492 | 0.0648 | 0.5493 | 0.0649 | 0.0077 | 0.0017 | 0.0583 | 0.0153 |
| Hubei | 2022 | 0.5991 | 0.0718 | 0.5992 | 0.0719 | 0.0084 | 0.0019 | 0.0636 | 0.0170 |
| Hubei | 2023 | 0.3858 | 0.0713 | 0.3859 | 0.0714 | 0.0054 | 0.0019 | 0.0410 | 0.0169 |
| Hunan | 2014 | 0.2572 | 0.0239 | 0.2573 | 0.0240 | 0.0036 | 0.0006 | 0.0273 | 0.0057 |
| Hunan | 2015 | 0.2587 | 0.0227 | 0.2588 | 0.0228 | 0.0036 | 0.0006 | 0.0275 | 0.0054 |
| Hunan | 2016 | 0.2708 | 0.0203 | 0.2709 | 0.0204 | 0.0038 | 0.0006 | 0.0288 | 0.0048 |
| Hunan | 2017 | 0.2950 | 0.0279 | 0.2951 | 0.0280 | 0.0042 | 0.0008 | 0.0313 | 0.0066 |
| Hunan | 2018 | 0.2965 | 0.0361 | 0.2966 | 0.0362 | 0.0042 | 0.0010 | 0.0315 | 0.0086 |
| Hunan | 2019 | 0.3162 | 0.0489 | 0.3163 | 0.0490 | 0.0045 | 0.0013 | 0.0336 | 0.0116 |
| Hunan | 2020 | 0.3177 | 0.0550 | 0.3178 | 0.0551 | 0.0045 | 0.0015 | 0.0337 | 0.0130 |
| Hunan | 2021 | 0.3374 | 0.0722 | 0.3375 | 0.0723 | 0.0048 | 0.0019 | 0.0358 | 0.0171 |
| Hunan | 2022 | 0.3389 | 0.0819 | 0.3390 | 0.0820 | 0.0048 | 0.0022 | 0.0360 | 0.0194 |
| Hunan | 2023 | 0.2542 | 0.0685 | 0.2543 | 0.0686 | 0.0036 | 0.0018 | 0.0270 | 0.0162 |
| Guangdong | 2014 | 0.1589 | 0.8413 | 0.1590 | 0.8414 | 0.0022 | 0.0226 | 0.0169 | 0.1990 |
| Guangdong | 2015 | 0.1649 | 0.7991 | 0.1650 | 0.7992 | 0.0023 | 0.0214 | 0.0175 | 0.1890 |
| Guangdong | 2016 | 0.1679 | 0.7465 | 0.1680 | 0.7466 | 0.0024 | 0.0200 | 0.0178 | 0.1766 |
| Guangdong | 2017 | 0.1755 | 0.7865 | 0.1756 | 0.7866 | 0.0025 | 0.0211 | 0.0186 | 0.1861 |
| Guangdong | 2018 | 0.1770 | 0.8475 | 0.1771 | 0.8476 | 0.0025 | 0.0227 | 0.0188 | 0.2005 |
| Guangdong | 2019 | 0.1710 | 0.8101 | 0.1711 | 0.8102 | 0.0024 | 0.0217 | 0.0182 | 0.1916 |
| Guangdong | 2020 | 0.1785 | 0.8003 | 0.1786 | 0.8004 | 0.0025 | 0.0215 | 0.0190 | 0.1893 |
| Guangdong | 2021 | 0.2390 | 1.0000 | 0.2391 | 1.0001 | 0.0034 | 0.0268 | 0.0254 | 0.2366 |
| Guangdong | 2022 | 0.2663 | 0.9734 | 0.2664 | 0.9735 | 0.0038 | 0.0261 | 0.0283 | 0.2303 |
| Guangdong | 2023 | 0.2345 | 0.9221 | 0.2346 | 0.9222 | 0.0033 | 0.0247 | 0.0249 | 0.2181 |
| Guangxi | 2014 | 0.1074 | 0.0315 | 0.1075 | 0.0316 | 0.0015 | 0.0009 | 0.0114 | 0.0075 |
| Guangxi | 2015 | 0.1210 | 0.0397 | 0.1211 | 0.0398 | 0.0017 | 0.0011 | 0.0129 | 0.0094 |
| Guangxi | 2016 | 0.1256 | 0.0370 | 0.1257 | 0.0371 | 0.0018 | 0.0010 | 0.0133 | 0.0088 |
| Guangxi | 2017 | 0.1346 | 0.0445 | 0.1347 | 0.0446 | 0.0019 | 0.0012 | 0.0143 | 0.0105 |
| Guangxi | 2018 | 0.1589 | 0.0485 | 0.1590 | 0.0486 | 0.0022 | 0.0013 | 0.0169 | 0.0115 |
| Guangxi | 2019 | 0.1831 | 0.0531 | 0.1832 | 0.0532 | 0.0026 | 0.0014 | 0.0195 | 0.0126 |
| Guangxi | 2020 | 0.2542 | 0.0548 | 0.2543 | 0.0549 | 0.0036 | 0.0015 | 0.0270 | 0.0130 |
| Guangxi | 2021 | 0.2481 | 0.0714 | 0.2482 | 0.0715 | 0.0035 | 0.0019 | 0.0264 | 0.0169 |
| Guangxi | 2022 | 0.2451 | 0.0748 | 0.2452 | 0.0749 | 0.0035 | 0.0020 | 0.0260 | 0.0177 |
| Guangxi | 2023 | 0.1876 | 0.0765 | 0.1877 | 0.0766 | 0.0026 | 0.0021 | 0.0199 | 0.0181 |
| Hainan | 2014 | 0.1543 | 0.0122 | 0.1544 | 0.0123 | 0.0022 | 0.0003 | 0.0164 | 0.0029 |
| Hainan | 2015 | 0.1815 | 0.0107 | 0.1816 | 0.0108 | 0.0026 | 0.0003 | 0.0193 | 0.0026 |
| Hainan | 2016 | 0.1831 | 0.0086 | 0.1832 | 0.0087 | 0.0026 | 0.0002 | 0.0195 | 0.0021 |
| Hainan | 2017 | 0.1937 | 0.0079 | 0.1938 | 0.0080 | 0.0027 | 0.0002 | 0.0206 | 0.0019 |
| Hainan | 2018 | 0.1921 | 0.0097 | 0.1922 | 0.0098 | 0.0027 | 0.0003 | 0.0204 | 0.0023 |
| Hainan | 2019 | 0.1921 | 0.0100 | 0.1922 | 0.0101 | 0.0027 | 0.0003 | 0.0204 | 0.0024 |
| Hainan | 2020 | 0.2088 | 0.0104 | 0.2089 | 0.0105 | 0.0029 | 0.0003 | 0.0222 | 0.0025 |
| Hainan | 2021 | 0.2496 | 0.0176 | 0.2497 | 0.0177 | 0.0035 | 0.0005 | 0.0265 | 0.0042 |
| Hainan | 2022 | 0.2073 | 0.0232 | 0.2074 | 0.0233 | 0.0029 | 0.0006 | 0.0220 | 0.0055 |
| Hainan | 2023 | 0.1740 | 0.0255 | 0.1741 | 0.0256 | 0.0025 | 0.0007 | 0.0185 | 0.0061 |
| Chongqing | 2014 | 0.1120 | 0.0744 | 0.1121 | 0.0745 | 0.0016 | 0.0020 | 0.0119 | 0.0176 |
| Chongqing | 2015 | 0.1120 | 0.0580 | 0.1121 | 0.0581 | 0.0016 | 0.0016 | 0.0119 | 0.0137 |
| Chongqing | 2016 | 0.1180 | 0.0488 | 0.1181 | 0.0489 | 0.0017 | 0.0013 | 0.0125 | 0.0116 |
| Chongqing | 2017 | 0.1362 | 0.0518 | 0.1363 | 0.0519 | 0.0019 | 0.0014 | 0.0145 | 0.0123 |
| Chongqing | 2018 | 0.1452 | 0.0615 | 0.1453 | 0.0616 | 0.0021 | 0.0017 | 0.0154 | 0.0146 |
| Chongqing | 2019 | 0.1513 | 0.0654 | 0.1514 | 0.0655 | 0.0021 | 0.0018 | 0.0161 | 0.0155 |
| Chongqing | 2020 | 0.1528 | 0.0734 | 0.1529 | 0.0735 | 0.0022 | 0.0020 | 0.0162 | 0.0174 |
| Chongqing | 2021 | 0.1619 | 0.0966 | 0.1620 | 0.0967 | 0.0023 | 0.0026 | 0.0172 | 0.0229 |
| Chongqing | 2022 | 0.1906 | 0.0951 | 0.1907 | 0.0952 | 0.0027 | 0.0026 | 0.0203 | 0.0225 |
| Chongqing | 2023 | 0.1543 | 0.0791 | 0.1544 | 0.0792 | 0.0022 | 0.0021 | 0.0164 | 0.0187 |
| Sichuan | 2014 | 0.3056 | 0.0546 | 0.3057 | 0.0547 | 0.0043 | 0.0015 | 0.0325 | 0.0130 |
| Sichuan | 2015 | 0.3343 | 0.0398 | 0.3344 | 0.0399 | 0.0047 | 0.0011 | 0.0355 | 0.0094 |
| Sichuan | 2016 | 0.3555 | 0.0383 | 0.3556 | 0.0384 | 0.0050 | 0.0010 | 0.0378 | 0.0091 |
| Sichuan | 2017 | 0.3797 | 0.0530 | 0.3798 | 0.0531 | 0.0054 | 0.0014 | 0.0403 | 0.0126 |
| Sichuan | 2018 | 0.3752 | 0.0701 | 0.3753 | 0.0702 | 0.0053 | 0.0019 | 0.0399 | 0.0166 |
| Sichuan | 2019 | 0.3812 | 0.0767 | 0.3813 | 0.0768 | 0.0054 | 0.0021 | 0.0405 | 0.0182 |
| Sichuan | 2020 | 0.3843 | 0.0911 | 0.3844 | 0.0912 | 0.0054 | 0.0025 | 0.0408 | 0.0216 |
| Sichuan | 2021 | 0.3979 | 0.1149 | 0.3980 | 0.1150 | 0.0056 | 0.0031 | 0.0423 | 0.0272 |
| Sichuan | 2022 | 0.4720 | 0.1176 | 0.4721 | 0.1177 | 0.0067 | 0.0032 | 0.0501 | 0.0278 |
| Sichuan | 2023 | 0.4055 | 0.1060 | 0.4056 | 0.1061 | 0.0057 | 0.0028 | 0.0431 | 0.0251 |
| Guizhou | 2014 | 0.1059 | 0.0082 | 0.1060 | 0.0083 | 0.0015 | 0.0002 | 0.0113 | 0.0020 |
| Guizhou | 2015 | 0.1044 | 0.0093 | 0.1045 | 0.0094 | 0.0015 | 0.0003 | 0.0111 | 0.0022 |
| Guizhou | 2016 | 0.1044 | 0.0042 | 0.1045 | 0.0043 | 0.0015 | 0.0001 | 0.0111 | 0.0010 |
| Guizhou | 2017 | 0.1210 | 0.0061 | 0.1211 | 0.0062 | 0.0017 | 0.0002 | 0.0129 | 0.0015 |
| Guizhou | 2018 | 0.1316 | 0.0057 | 0.1317 | 0.0058 | 0.0019 | 0.0002 | 0.0140 | 0.0014 |
| Guizhou | 2019 | 0.1316 | 0.0049 | 0.1317 | 0.0050 | 0.0019 | 0.0001 | 0.0140 | 0.0012 |
| Guizhou | 2020 | 0.1331 | 0.0059 | 0.1332 | 0.0060 | 0.0019 | 0.0002 | 0.0142 | 0.0014 |
| Guizhou | 2021 | 0.1407 | 0.0077 | 0.1408 | 0.0078 | 0.0020 | 0.0002 | 0.0150 | 0.0018 |
| Guizhou | 2022 | 0.1815 | 0.0082 | 0.1816 | 0.0083 | 0.0026 | 0.0002 | 0.0193 | 0.0020 |
| Guizhou | 2023 | 0.1785 | 0.0082 | 0.1786 | 0.0083 | 0.0025 | 0.0002 | 0.0190 | 0.0020 |
| Yunnan | 2014 | 0.1241 | 0.0229 | 0.1242 | 0.0230 | 0.0018 | 0.0006 | 0.0132 | 0.0054 |
| Yunnan | 2015 | 0.1241 | 0.0189 | 0.1242 | 0.0190 | 0.0018 | 0.0005 | 0.0132 | 0.0045 |
| Yunnan | 2016 | 0.1301 | 0.0153 | 0.1302 | 0.0154 | 0.0018 | 0.0004 | 0.0138 | 0.0037 |
| Yunnan | 2017 | 0.1831 | 0.0181 | 0.1832 | 0.0182 | 0.0026 | 0.0005 | 0.0195 | 0.0043 |
| Yunnan | 2018 | 0.2012 | 0.0231 | 0.2013 | 0.0232 | 0.0028 | 0.0006 | 0.0214 | 0.0055 |
| Yunnan | 2019 | 0.2058 | 0.0261 | 0.2059 | 0.0262 | 0.0029 | 0.0007 | 0.0219 | 0.0062 |
| Yunnan | 2020 | 0.2375 | 0.0304 | 0.2376 | 0.0305 | 0.0034 | 0.0008 | 0.0252 | 0.0072 |
| Yunnan | 2021 | 0.2436 | 0.0378 | 0.2437 | 0.0379 | 0.0034 | 0.0010 | 0.0259 | 0.0090 |
| Yunnan | 2022 | 0.2632 | 0.0377 | 0.2633 | 0.0378 | 0.0037 | 0.0010 | 0.0280 | 0.0090 |
| Yunnan | 2023 | 0.2103 | 0.0285 | 0.2104 | 0.0286 | 0.0030 | 0.0008 | 0.0223 | 0.0068 |
| Tibet | 2014 | 0.0000 | 0.0015 | 0.0001 | 0.0016 | 0.0000 | 0.0000 | 0.0000 | 0.0004 |
| Tibet | 2015 | 0.0045 | 0.0005 | 0.0046 | 0.0006 | 0.0001 | 0.0000 | 0.0005 | 0.0001 |
| Tibet | 2016 | 0.0045 | 0.0004 | 0.0046 | 0.0005 | 0.0001 | 0.0000 | 0.0005 | 0.0001 |
| Tibet | 2017 | 0.0045 | 0.0004 | 0.0046 | 0.0005 | 0.0001 | 0.0000 | 0.0005 | 0.0001 |
| Tibet | 2018 | 0.0045 | 0.0003 | 0.0046 | 0.0004 | 0.0001 | 0.0000 | 0.0005 | 0.0001 |
| Tibet | 2019 | 0.0045 | 0.0003 | 0.0046 | 0.0004 | 0.0001 | 0.0000 | 0.0005 | 0.0001 |
| Tibet | 2020 | 0.0061 | 0.0000 | 0.0062 | 0.0001 | 0.0001 | 0.0000 | 0.0007 | 0.0000 |
| Tibet | 2021 | 0.0136 | 0.0002 | 0.0137 | 0.0003 | 0.0002 | 0.0000 | 0.0015 | 0.0001 |
| Tibet | 2022 | 0.0166 | 0.0003 | 0.0167 | 0.0004 | 0.0002 | 0.0000 | 0.0018 | 0.0001 |
| Tibet | 2023 | 0.0166 | 0.0010 | 0.0167 | 0.0011 | 0.0002 | 0.0000 | 0.0018 | 0.0003 |
| Shaanxi | 2014 | 0.3540 | 0.0212 | 0.3541 | 0.0213 | 0.0050 | 0.0006 | 0.0376 | 0.0050 |
| Shaanxi | 2015 | 0.3707 | 0.0236 | 0.3708 | 0.0237 | 0.0052 | 0.0006 | 0.0394 | 0.0056 |
| Shaanxi | 2016 | 0.4085 | 0.0232 | 0.4086 | 0.0233 | 0.0058 | 0.0006 | 0.0434 | 0.0055 |
| Shaanxi | 2017 | 0.4206 | 0.0311 | 0.4207 | 0.0312 | 0.0059 | 0.0008 | 0.0447 | 0.0074 |
| Shaanxi | 2018 | 0.4387 | 0.0414 | 0.4388 | 0.0415 | 0.0062 | 0.0011 | 0.0466 | 0.0098 |
| Shaanxi | 2019 | 0.4387 | 0.0397 | 0.4388 | 0.0398 | 0.0062 | 0.0011 | 0.0466 | 0.0094 |
| Shaanxi | 2020 | 0.4614 | 0.0424 | 0.4615 | 0.0425 | 0.0065 | 0.0011 | 0.0490 | 0.0101 |
| Shaanxi | 2021 | 0.4660 | 0.0573 | 0.4661 | 0.0574 | 0.0066 | 0.0015 | 0.0495 | 0.0136 |
| Shaanxi | 2022 | 0.4796 | 0.0556 | 0.4797 | 0.0557 | 0.0068 | 0.0015 | 0.0509 | 0.0132 |
| Shaanxi | 2023 | 0.3661 | 0.0447 | 0.3662 | 0.0448 | 0.0052 | 0.0012 | 0.0389 | 0.0106 |
| Gansu | 2014 | 0.2163 | 0.0065 | 0.2164 | 0.0066 | 0.0031 | 0.0002 | 0.0230 | 0.0016 |
| Gansu | 2015 | 0.2209 | 0.0060 | 0.2210 | 0.0061 | 0.0031 | 0.0002 | 0.0235 | 0.0014 |
| Gansu | 2016 | 0.2239 | 0.0051 | 0.2240 | 0.0052 | 0.0032 | 0.0001 | 0.0238 | 0.0012 |
| Gansu | 2017 | 0.3026 | 0.0037 | 0.3027 | 0.0038 | 0.0043 | 0.0001 | 0.0321 | 0.0009 |
| Gansu | 2018 | 0.3192 | 0.0045 | 0.3193 | 0.0046 | 0.0045 | 0.0001 | 0.0339 | 0.0011 |
| Gansu | 2019 | 0.3328 | 0.0041 | 0.3329 | 0.0042 | 0.0047 | 0.0001 | 0.0354 | 0.0010 |
| Gansu | 2020 | 0.3359 | 0.0041 | 0.3360 | 0.0042 | 0.0047 | 0.0001 | 0.0357 | 0.0010 |
| Gansu | 2021 | 0.3389 | 0.0057 | 0.3390 | 0.0058 | 0.0048 | 0.0002 | 0.0360 | 0.0014 |
| Gansu | 2022 | 0.3419 | 0.0065 | 0.3420 | 0.0066 | 0.0048 | 0.0002 | 0.0363 | 0.0016 |
| Gansu | 2023 | 0.2874 | 0.0052 | 0.2875 | 0.0053 | 0.0041 | 0.0001 | 0.0305 | 0.0013 |
| Qinghai | 2014 | 0.0272 | 0.0011 | 0.0273 | 0.0012 | 0.0004 | 0.0000 | 0.0029 | 0.0003 |
| Qinghai | 2015 | 0.0287 | 0.0013 | 0.0288 | 0.0014 | 0.0004 | 0.0000 | 0.0031 | 0.0003 |
| Qinghai | 2016 | 0.0287 | 0.0010 | 0.0288 | 0.0011 | 0.0004 | 0.0000 | 0.0031 | 0.0003 |
| Qinghai | 2017 | 0.0287 | 0.0003 | 0.0288 | 0.0004 | 0.0004 | 0.0000 | 0.0031 | 0.0001 |
| Qinghai | 2018 | 0.0303 | 0.0003 | 0.0304 | 0.0004 | 0.0004 | 0.0000 | 0.0032 | 0.0001 |
| Qinghai | 2019 | 0.0303 | 0.0002 | 0.0304 | 0.0003 | 0.0004 | 0.0000 | 0.0032 | 0.0001 |
| Qinghai | 2020 | 0.0303 | 0.0000 | 0.0304 | 0.0001 | 0.0004 | 0.0000 | 0.0032 | 0.0000 |
| Qinghai | 2021 | 0.0303 | 0.0001 | 0.0304 | 0.0002 | 0.0004 | 0.0000 | 0.0032 | 0.0001 |
| Qinghai | 2022 | 0.0303 | 0.0002 | 0.0304 | 0.0003 | 0.0004 | 0.0000 | 0.0032 | 0.0001 |
| Qinghai | 2023 | 0.0303 | 0.0003 | 0.0304 | 0.0004 | 0.0004 | 0.0000 | 0.0032 | 0.0001 |
| Ningxia | 2014 | 0.0121 | 0.0040 | 0.0122 | 0.0041 | 0.0002 | 0.0001 | 0.0013 | 0.0010 |
| Ningxia | 2015 | 0.0121 | 0.0027 | 0.0122 | 0.0028 | 0.0002 | 0.0001 | 0.0013 | 0.0007 |
| Ningxia | 2016 | 0.0136 | 0.0023 | 0.0137 | 0.0024 | 0.0002 | 0.0001 | 0.0015 | 0.0006 |
| Ningxia | 2017 | 0.0756 | 0.0037 | 0.0757 | 0.0038 | 0.0011 | 0.0001 | 0.0080 | 0.0009 |
| Ningxia | 2018 | 0.0756 | 0.0027 | 0.0757 | 0.0028 | 0.0011 | 0.0001 | 0.0080 | 0.0007 |
| Ningxia | 2019 | 0.0772 | 0.0025 | 0.0773 | 0.0026 | 0.0011 | 0.0001 | 0.0082 | 0.0006 |
| Ningxia | 2020 | 0.0756 | 0.0012 | 0.0757 | 0.0013 | 0.0011 | 0.0000 | 0.0080 | 0.0003 |
| Ningxia | 2021 | 0.0908 | 0.0024 | 0.0909 | 0.0025 | 0.0013 | 0.0001 | 0.0097 | 0.0006 |
| Ningxia | 2022 | 0.0908 | 0.0023 | 0.0909 | 0.0024 | 0.0013 | 0.0001 | 0.0097 | 0.0006 |
| Ningxia | 2023 | 0.0726 | 0.0021 | 0.0727 | 0.0022 | 0.0010 | 0.0001 | 0.0077 | 0.0005 |
| Xinjiang | 2014 | 0.1180 | 0.0214 | 0.1181 | 0.0215 | 0.0017 | 0.0006 | 0.0125 | 0.0051 |
| Xinjiang | 2015 | 0.1241 | 0.0151 | 0.1242 | 0.0152 | 0.0018 | 0.0004 | 0.0132 | 0.0036 |
| Xinjiang | 2016 | 0.1301 | 0.0136 | 0.1302 | 0.0137 | 0.0018 | 0.0004 | 0.0138 | 0.0032 |
| Xinjiang | 2017 | 0.1301 | 0.0159 | 0.1302 | 0.0160 | 0.0018 | 0.0004 | 0.0138 | 0.0038 |
| Xinjiang | 2018 | 0.1316 | 0.0154 | 0.1317 | 0.0155 | 0.0019 | 0.0004 | 0.0140 | 0.0037 |
| Xinjiang | 2019 | 0.1301 | 0.0183 | 0.1302 | 0.0184 | 0.0018 | 0.0005 | 0.0138 | 0.0044 |
| Xinjiang | 2020 | 0.1165 | 0.0165 | 0.1166 | 0.0166 | 0.0016 | 0.0004 | 0.0124 | 0.0039 |
| Xinjiang | 2021 | 0.1120 | 0.0188 | 0.1121 | 0.0189 | 0.0016 | 0.0005 | 0.0119 | 0.0045 |
| Xinjiang | 2022 | 0.2496 | 0.0284 | 0.2497 | 0.0285 | 0.0035 | 0.0008 | 0.0265 | 0.0067 |
| Xinjiang | 2023 | 0.1422 | 0.0394 | 0.1423 | 0.0395 | 0.0020 | 0.0011 | 0.0151 | 0.0093 |
